# Prehabilitation for lumbar spinal stenosis: understanding mechanisms and contexts for enhanced engagement—a realist review

**DOI:** 10.1093/ageing/afaf311

**Published:** 2025-10-24

**Authors:** Rebecca Hunter, Andrew Booth, Sarah (Sallie) Lamb, Esther Williamson, Paul Hendrick, Opinder Sahota, Bethan E Phillips, Judith Fitch, Lianne Wood

**Affiliations:** Department of Public Health and Sports Sciences, University of Exeter, Medical School Building, St Luke’s Campus, Exeter EX4 4QJ, UK; School of Health and Related Research (ScHARR), The University of Sheffield, Sheffield, England, UK; Department of Public Health and Sports Sciences, University of Exeter, Medical School Building, St Luke’s Campus, Exeter EX4 4QJ, UK; Department of Public Health and Sports Sciences, University of Exeter, Medical School Building, St Luke’s Campus, Exeter EX4 4QJ, UK; Nuffield Department of Orthopaedics, Rheumatology and Musculoskeletal Sciences, University of Oxford, Botnar Research Centre, Old Road Oxford, Oxford, Oxfordshire OX3 7LD, UK; Faculty of Medicine and Health Sciences, University of Nottingham, Nottingham, UK; Department of Health Care of Older People (HCOP), Queen’s Medical Centre (QMC), B Floor, South Block, Nottingham, Derby NG72UH, UK; MRC-ARUK Centre for Musculoskeletal Ageing Research, University of Nottingham, Royal Derby Hospital Centre, Derby DE22 3DT, UK; Independent Patient and Public Involvement (PPI) Contributor, Beverley, UK; Department of Public Health and Sports Sciences, University of Exeter, Medical School Building, St Luke’s Campus, Exeter EX4 4QJ, UK

**Keywords:** lumbar spinal stenosis, neurogenic claudication, prehabilitation, spinal surgery, older people

## Abstract

**Background:**

Neurogenic claudication (NC) due to lumbar spinal stenosis is the most common reason for spinal surgery in older adults. Prehabilitation may improve outcomes and reduce costs, but current evidence is conflicting. It remains unclear who benefits most, which mechanisms optimise outcomes and what outcomes matter to patients. This review aimed to develop a programme theory explaining what works, for whom, how and in what contexts for prehabilitation of NC surgical candidates.

**Methods:**

An initial programme theory, comprising context-mechanism-outcome configurations (CMOCs), was developed through iterative mapping and consultation with experts (*n* = 6) and patients (*n* = 7). This theory was refined via two systematic literature searches and further stakeholder feedback. Studies were assessed for relevance, richness and rigour. Data were holistically coded using abductive and retroductive reasoning to create causal maps, which informed CMOC refinement.

**Results:**

From 1422 records, 67 papers were included. The final programme theory included 14 CMOCs focused on patient engagement, a priority identified through patient consultation. Engagement was contingent on clear, consistent communication and addressing misconceptions among both patients and professionals. A shared understanding increased perceived value and avoided missed opportunities for preparation. Personalisation and collaborative goal-setting enhanced ownership and motivation. Ongoing support—*via* healthcare professional contact and peer input—helped counteract anxiety and feelings of abandonment during the surgical wait.

**Conclusions:**

Engagement with prehabilitation for NC can be improved through clear communication, tailored interventions and sustained support. Further research is needed to test whether theory-informed programmes improve outcomes in this population.

## Key Points

- Prehabilitation for older adults with neurogenic claudication must balance personalisation with fidelity and scalability.
- Ongoing support during the wait for surgery is vital to maintain engagement and reduce feelings of abandonment.
- A clear programme theory should integrate individual needs and system-level enablers like communication and tailoring.
- Further research is needed to define core components for safe, acceptable and effective real-world delivery.

## Background

An estimated 103 million older adults worldwide are affected by lumbar spinal stenosis (LSS) annually [1]. When conservative management fails, surgery may be considered. LSS surgery for symptomatic neurogenic claudication (NC) is the most common spinal surgery among older adults [2], and with a globally ageing population, demand is expected to rise. In the USA, LSS surgery has seen the fastest growth among lumbar spine procedures [3], a trend mirrored globally with a rise in minimally invasive LSS surgeries aimed at reducing recovery time and surgical risk [4]. Demand on healthcare services has been further exacerbated by the COVID-19 pandemic: the suspension of elective surgeries, including spinal procedures, created a substantial backlog [5], leading to longer waiting times for non-urgent surgeries [6].

LSS surgery can be physically and psychologically demanding for patients. Studies have shown that longer waiting times for LSS surgery can negatively affect post-operative pain, physical function and recovery times [7–9]. Although decompression surgery for symptomatic LSS is considered routine [10], it still imposes significant physiological stress on the body, highlighting the need for patients to be adequately prepared [11]. Prehabilitation is a pre-operative intervention designed to enhance a patient’s functional capacity, mitigate modifiable risk factors and address the psychological stress of surgery and pre-operative anxiety [12]. Expert guidance suggests a multimodal approach to prehabilitation encompassing physical exercise, nutritional guidance and psychological support to optimise patient and surgical outcomes [12, 13]. Given the potential negative impact of surgical wait times, further investigation of prehabilitation as a strategy to optimise readiness for LSS surgery, maintain function during the waiting period and improve post-operative outcomes is warranted.

Although evidence exists to support the effectiveness of prehabilitation programmes for improving surgical outcomes in cancer patients, the evidence for prehabilitation in LSS surgery is conflicting. A meta-analysis by Janssen *et al*. [14], found no additional benefit from cognitive-behavioural and exercise-based prehabilitation for LSS above usual care. In contrast, Nielsen *et al*. [15], studying spinal patients more broadly, reported improved outcomes, including shorter hospital stays, from a prehabilitation programme combining physical therapy and protein supplementation. Marchand *et al*. [16] found that exercise-based prehabilitation improved pre-operative outcomes (pain intensity, disability, strength and function), but most effects were not sustained after surgery. These inconsistencies highlight the need to look beyond traditional efficacy studies and instead explore what works, for whom and why—an approach that may help explain the mixed results and guide the design of more effective, tailored prehabilitation programmes. Realist reviews are designed to explore how and why complex interventions work, for whom and under what conditions, by examining the interplay between context, mechanisms and outcomes [17].

The aim of this realist review was to develop a programme theory explaining the factors influencing prehabilitation outcomes of NC surgical candidates. Our research questions were:

1. What are the mechanisms through which prehabilitation interventions for NC surgical candidates produce their effects?
2. What are the contextual factors that influence the effectiveness of prehabilitation interventions for NC surgical candidates?

## Methods

### Study design

A realist review was undertaken following Pawson’s methodological framework [18] and conducted in accordance with the Realist And Meta-narrative Evidence Synthesis: Evolving Standards (RAMESES) quality and reporting standards [19, 20] (see Supplementary Appendix S1). The study was registered with PROSPERO (CRD42024564345).

### Development and refinement of the programme theory

**Figure 1:**
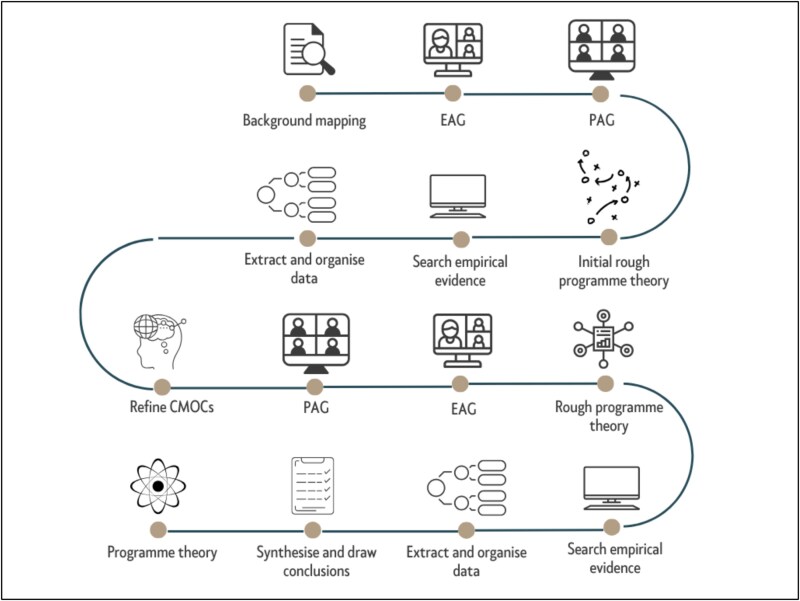
Stages of the realist review. EAG: Expert Advisory Group; PAG: Patient Advisory Group; CMOCs: context-mechanism-outcome configurations.

An initial programme theory was developed through background scoping (Google Scholar) and expert and patient advisory input. This theory was iteratively refined into context-mechanism-outcome (CMO) configurations *via* further literature searches and stakeholder engagement. Although presented linearly (Figure 1), the process was iterative. Guided by the patient advisory group (PAG), the review prioritised understanding patient engagement, focusing on behavioural and contextual factors, rather than detailed intervention components.

### Stakeholder consultations

This review was informed by two advisory groups, comprising patients with lived experience of LSS surgery and experts in LSS and prehabilitation. Four consultations were held at key stages to support the credibility and refinement of the CMO configurations (CMOCs, see Figure 1). Further details of the expert and PAGs are provided in Supplementary Appendix S2.

### Search strategy and selection criteria

A two-phase literature search was conducted to identify, refine and test CMOCs relevant to prehabilitation in LSS.

Phase 1: Comprehensive search.

In June 2024, a systematic search was carried out across four databases: MEDLINE via PubMed, CINAHL, Scopus and Web of Science (Science and Social Science Citation Indexes). The strategy focused on the core concepts of prehabilitation, spinal conditions and orthopaedic surgery. Searches were tailored for each database using appropriate thesaurus terms and syntax (see Supplementary Appendix S2 for the full search log). All results were imported into Rayyan (https://www.rayyan.ai) for screening. Searches were limited to English-language publications from January 2011 to June 2024.

Phase 2: Targeted CMOC testing.

To test and refine emerging CMOCs, a targeted search was conducted using two AI-assisted platforms—Scite (v0.4.5) and Undermind (v0.4.7)—in November 2024 (pilot) and February 2025. These tools enabled rapid evidence mapping through citation tracking and snowballing. Scite’s claim-analysis feature examined how concepts within each CMOC were cited and supported in the literature, while undermind generated topic-specific evidence summaries that highlighted key findings, barriers, future directions and relevant citations (see sample reports in Supplementary Appendices S4–S5).

### Study selection and appraisal

#### Phase 1: Selection

Title and abstract screening was conducted using a bespoke screening tool (Supplementary Appendix S6), scoring relevance on a 1–5 scale. Articles scoring three or higher were retained, resulting in 55 papers eligible for full-text screening (one was unobtainable). The remaining 54 underwent detailed appraisal using a bespoke Relevance, Richness and Rigour (RRR) tool (Supplementary Appendix S7), with each criterion scored as high, moderate, or low. Papers scoring low in all three domains were excluded. To ensure consistency, 20% of records were reviewed by a second researcher (LW), with disagreements resolved by consensus.

#### Phase 2: Selection

AI-generated outputs were screened for empirical relevance to each CMOC. Only primary qualitative or quantitative studies were included. When a relevant secondary source was identified, backward citation tracking was used to trace primary studies. All included papers were assessed using a tailored RRR tool (Supplementary Appendix S8) that evaluated each study’s relevance, explanatory power and rigour in relation to the CMOCs. A second researcher (L.W.) assessed 50% of excluded papers for each CMOC to verify initial judgements.

### Assessment of rigour

Rigour was assessed at both the document and data levels, guided by recommendations from Dada *et al*. [21]. Judgements were made based on the credibility of the source and the trustworthiness of the methods used to generate the data. The Crowe Critical Appraisal Tool (CCAT v1.4) was used as an additional framework to support these assessments [22].

### Data extraction

Data extraction was conducted by a single researcher (R.H.), with metadata—publication year, study country, surgical condition, study design and participant characteristics—recorded in Microsoft Excel. Qualitative data were annotated using a bespoke Microsoft Word extraction form (see Supplementary Appendix S9), including interpretive notes to identify relevant data for CMOC development.

### Analysis and synthesis

An iterative, theory-driven approach underpinned data synthesis. Using abductive and retroductive reasoning [23], emerging data were coded holistically—reflective annotations capturing broader meanings and connections across studies—were developed to create and test causal explanations. These codes were mapped in Miro (2024) using a mind-mapping template, with annotated arrows illustrating relationships. As data accumulated, maps were refined, generating new insights. Team discussions and expert and PAG feedback consolidated these insights, refining the programme theory and finalising the CMOCs.

## Results

### Document characteristics

**Figure 2:**
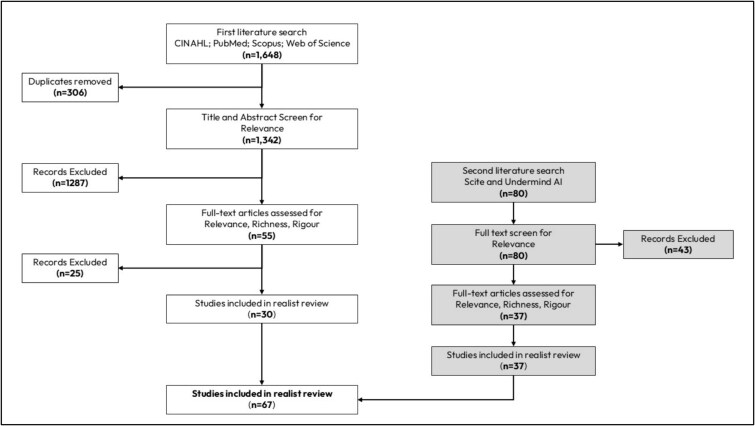
Flow of studies in the review.

Sixty-seven papers published between 2015 and 2024 were included (see Figure 2). All were rated moderately (*n* = 37) or highly relevant (*n* = 30), with moderate (*n* = 17) or high (*n* = 50) rigour. Studies covered prehabilitation for major elective abdominal (*n* = 4), general (*n* = 4), knee arthroplasty (*n* = 1), general orthopaedic (*n* = 2), orthopaedic/cardiac (*n* = 1), orthopaedic/neurological (*n* = 1), lumbar (*n* = 24), spinal (*n* = 6; cervical, thoracic, lumbar) and cancer-related surgeries (*n* = 23). One non-surgical study of rheumatic pain clinic patients also offered relevant insights. A summary of the included documents and their key characteristics is provided in Table 1.

**Table 1:** Table of characteristics of included documents

Study	Year	Study design	Participants	Condition	Aim of study	Country	Relevance	Richness	Rigour	CMOCs
Alsaif [24]	2023	Qualitative study	(a) Adult patients listed for surgery and attended preoperative rehabilitation (n = 7); (b) Adult patients listed for surgery but did not attend rehabilitation (n = 5), and; (c) HCPs involved in the delivery of rehabilitation (n = 8)	Lumbar discectomy	To develop an understanding of patient and healthcare provider (HCP) experiences, perspectives and preferences of preoperative LD rehabilitation, including why patients do not attend	UK	Moderate	High	High	5, 6, 10
Arguisuelas [25]	2024	Randomised Controlled Trial—Protocol	100 patients 18–80 y.o; Experimental group (n = 50); Control group (n = 50)	Lumbar radiculopathy (elective surgery)	To evaluate the effectiveness of a multicomponent prehabilitation programme administered through educational videos versus another programme based on written exercise recommendations, in patients scheduled for lumbar radiculopathy surgery.	Spain	Moderate	High	High	7, 9
Bakaa [26]	2022	Pilot Randomised Controlled Trial (mixed methods)	60 patients; 8-week (eHealth) prehabilitation programme (n = 30) or minimal intervention (n = 30). Plus semi-structured interviews (n = 12–15)	Lumbar spinal stenosis (LSS)	To evaluate the feasibility of an eHealth prehabilitation programme for individuals undergoing LSS surgery, and an embedded longitudinal qualitative study explores the perioperative patient experience and recovery trajectory	Canada	High	High	High	10, 11, 12
Barberan-Garcia [27]	2018	RCT	125 patients (n = 63 control; 20% female; mean 71 y.o (10); n = 62 intervention; 32% female, mean 71 y.o (11).	Abdominal	To assess the impact of a personalised prehabilitation on postoperative complications in high-risk patients undergoing elective major abdominal surgery	Spain	Moderate	Moderate	High	2
Barnes [28]	2023	Qualitative study	15 participants—mean age 72 y.o (60–85); 8 female (53%)	Cancer (general)	To qualitatively assess the barriers and facilitators to participating in exercise prehabilitation from the perspective of older people with frailty participating in the intervention arm of a randomised trial.	Canada	High	High	High	2, 9, 13
Beck [29]	2021a	Mixed methods	53/73 patients (73%) returned a completed leaflet. 7 men(13%) and 46 women (87%) with a mean age of 62 y.o (95% CI: 59–65); 5 patient interview – 3 women (aged 51–52 y.o) 2 men (51 & 65 y.o)	Abdominal	To investigate what patients with cancer who were due to undergo major abdominal surgery were actually able to do when provided with preoperative, home-based, multimodal recommendations presented in a leaflet.	Denmark	High	High	Moderate	2, 11
Beck [30]	2021b	Qualitative study	79 patients. 31 interviews (n = 19 female); mean age 60 y.o53 activity registrations (n = 46 female);mean age 62	Cancer surgery (abdominal)	To understand perspectives on and acceptability of prehabilitation among patients undergoing complex abdominal cancer surgery.	Denmark	High	Moderate	High	1, 3, 8, 11
Beck [31]	2022	Qualitative study	16 patients (n = 4 female); median age 58 y.o	Cancer (colorectal or ovarian)	To investigate the experiences, thoughts, and feelings that underlie and influence prehabilitation among cancer patients due to undergo major abdominal surgery	Denmark	High	Moderate	High	1, 9, 10, 11

*(continued)*

**Table 1:** Continued

Study	Year	Study design	Participants	Condition	Aim of study	Country	Relevance	Richness	Rigour	CMOCs
Bingham [32]	2023	Qualitative study	33 interviews (n = 24 interdisciplinary professionals;19 female; n = 9 patients, age range 46–60 n = 2, 61–75 n = 6, 76+ n = 1; female n = 3)	Cancer surgery	To explore mechanisms promoting feasibility and acceptability of a multi-modal cancer prehabilitation program-me from patients’ and professionals’ perspectives exploring planning, development and implementation phases	UK	High	High	High	1, 6, 12, 13
Boukili [33]	2022	Pilot study	60 patients (n = 34 women); median age 62.5 y.o.	Abdominal	To evaluate the feasibility and safety of a prehabilitation programme before major abdominal surgery.	France	High	Moderate	Moderate	6, 13, 14
Brahmbhatt [34]	2024	Mixed methods	72 participants—all female; Intervention (n = 35); mean age 57.4 ± 11.9; Control (n = 37) mean age 54. 0 ± 10.7	Cancer (breast)	To investigate the feasibility, effectiveness, and acceptability of a multimodal prehabilitation programme for women with breast cancer undergoing neoadjuvant chemotherapy (NACT).	Canada	Moderate	Low	Moderate	13
Briguglio [35]	2022	Perspective article	Not applicable	Elective orthopaedic surgery (general)	To present how a Hazard Analysis and Critical Control Point (HACCP)-derived methodology could be used to manage preoperative nutritional and physical risks (e.g. malnutrition, sarcopenia) and improve optimisation before orthopaedic surgery.	UK	Moderate	Moderate	Moderate	8, 13
Bruns [36]	2019	Feasibility study	Fourteen patients (median age 79, 5 males) participated. At baseline, 86% patients were physically impaired and 64% were at risk for malnourishment	Cancer (colorectal)	To assess the feasibility of Fit4SurgeryTV, an at home prehabilitation programme specifically designed for frail elderly with colorectal cancer (CRC)	Netherlands	Moderate	Low	Moderate	3
Burke [37]	2015	Phenomenological study	10 patients (female n = 7); Age 45 to 74 years (M = 58.2, SD = 7.7)	Cancer (rectal)	To explore the lived experience of patients with advanced rectal cancer as they attempted to adhere to a prescribed, hospital-based pre-surgical exercise programme	UK	Moderate	Moderate	High	10, 11
Carr [38]	2017	Qualitative study	32 patients, 16 men and 16 women, awaiting orthopaedic (n = 22) or cardiac surgery (n = 10). Age range: 43 to 89; median age of females 62.5 vs 59.5 males	Orthopaedic or Cardiac surgery	To understand experiences of wait time among patients awaiting scheduled orthopaedic or cardiac surgery	Canada	High	Moderate	High	1, 12, 13
Casanovas-Álvarez [39]	2024	Qualitative study	21 female patients (age range 37–68) participated in 2 focus groups	Cancer (breast)	To analyse the perceptions and experiences of patients with breast cancer (BC) who participated in a prehabilitation programme in order to improve future implementations of these much-needed programmes for patients with bc	Spain	High	High	Moderate	2, 6, 11, 12
Cooper [40]	2022	Qualitative study	Twenty-two participants (18 men, 4 women; aged 67 ± 8 years old) took part in a focus group discussion (n = 17) or a semi-structured interview (n = 5).	Cancer (general)	To identify factors influencing uptake, engagement and adherence of ChemoFit intervention	UK	High	High	High	2, 9, 12, 13
Delgado-López [41]	2019	Literature review	Seven papers	Degenerative lumbar spine disorder	A review of the literature on the usefulness of prehabilitation for degenerative spinal surgery	N/A	High	Moderate	Moderate	1

*(continued)*

**Table 1:** Continued

Study	Year	Study design	Participants	Condition	Aim of study	Country	Relevance	Richness	Rigour	CMOCs
Deslauriers [42]	2021	Qualitative study	26 participants (22 women and 4 men; mean age 54 ± 10 years).	Rheumatic conditions	To gain an in-depth understanding of perceptions and experiences of patients with rheumatic conditions regarding access to pain clinic services. The secondary objective was to identify possible solutions to improve this access according to patients’ perspectives	Canada	High	Moderate	High	13
Eastwood [43]	2019	Retrospective cohort study	206 patients; Cohort 1 patients who participated in preoperative multidisciplinary education (n = 103) 42 female, mean age + SD 58.98 + 14.28; Cohort 2 patients who opted out of the educational session (n = 103) 52 female, mean age + SD 58.98 + 12.61.	Elective spinal fusion	To determine if participation in a single preoperative multi-disciplinary educational session would result in reduced patient dissatisfaction with surgical expectations and if participation resulted in improvements in postsurgical pain, disability, and reductions in emergency room visits following surgery	Canada	Moderate	Moderate	High	5
Edwards [44]	2022	Cohort study	65 (43%) patients (mean age 57.4 years (SD 18.2), 58.8% female) comprised the Attend- POSE, and 85 (57%) DNA-POSE (mean age 54.9 years (SD 15.8), 65.8% female)	Spinal fusion surgery	To determine if a Pre-operative Spinal Education (POSE) program-me, specified using the Rehabilitation Treatment Specification System (RTSS) and designed to normalise expectations and reduce anxieties, was safe and reduced length of stay (LOS)	UK	Moderate	High	High	1, 2, 5, 7
Eubanks [45]	2023	Scoping review	23 studies assessing surgery for nonelective spine condition	Elective spine surgery (i.e Cervical, Thoracic, Lumbar spineincluded)	To identify and describe the current interventions used in preoperative programmes (‘prehabilitation’) for spine surgery	N/A	Moderate	High	High	5, 7, 14
Eubanks [46]	2024	Feasibility Study	Fifteen patients mean age 62 y.o (52–74 range); 53.3% female	Lumbar spinal stenosis	To determine the feasibility of delivery and acceptability by participants of a novel prehabilitation intervention for patients undergoing LSS surgery.	USA	High	Moderate	High	7,14
Francis-Coad [47]	2021	Qualitative study	18 patients (72% male (n = 13); 28% female (n = 5); 44% aged 70–79	General surgery	To explore patients’ experiences when preparing for and undergoing general surgery at a large tertiary hospital. Findings aimed to inform the development of a prehabilitation programme to empower patients to optimise their recovery and enhance their experience of general surgery	Australia	High	High	High	3, 6, 12, 13
Gillis [48]	2021	Qualitative study	20 patients interviews (mean age 62 y.o [SD13] yr)	General surgery	To determine how they prepared for surgery, their views on prehabilitation and how prehabilitation could be delivered to best meet patient needs.	Canada	High	High	High	2, 3, 5, 10, 12, 13
Gometz [49]	2018	Systematic review	Five papers based on three RCTs	Lumbar spinal surgery	To determine whether prehabilitation improves functional outcomes and reduces costs following spinal surgery.	N/A	Moderate	Moderate	High	7
Heil [50]	2022	Qualitative study	13 interviews five surgeons, three specialised nurses, three physical therapists, two dieticians.	Cancer (colorectal)	To explore perspectives of professionals involved in prehabilitation to gain understanding of barriers or facilitators to its implementation and to identify strategies to successful operationalisation of prehabilitation.	Netherlands	High	High	High	2, 5, 6, 9, 14

*(continued)*

**Table 1:** Continued

Study	Year	Study design	Participants	Condition	Aim of study	Country	Relevance	Richness	Rigour	CMOCs
Heldens [51]	2016	Feasibility study	9 patients (69.2%) completed the programme without adverse events. Four patients dropped out. Mean age 64.4 ± 10.9; male n = 8	Rectal Cancer	To determine the feasibility and preliminary effectiveness of a supervised outpatient physical exercise training programme during NACRT in these patients	Netherlands	Moderate	Low	High	9
Jandu [52]	2023	Qualitative study	54 participants; female n = 33 (61.1%); mean age 61.2 y.o (range 33–78)	Cancer (general)	To understand what a prehabilitation programme for cancer patients should include, based on the experiences and opinions of patients who have already gone through such a programme after their treatment.	UK	Moderate	Moderate	High	10, 12
Janssen [14]	2021	Systematic review with meta-analysis	15 studies; 11/15 included in meta-analysis	Degenerative lumbar spine disorder	To assess the effectiveness of prehabilitation in patients with degenerative disorders of the lumbar spine who are scheduled for spine surgery.	N/A	Moderate	Moderate	Moderate	2, 3
Kemani [53]	2020	Observational study	348 patients (age mean male = 48.2 ± 11.8; women 45.7 ± 12.3)	Lumbar spine	To evaluate change in fear of movement and the relationship of fear of movement and pain intensity to low back disability and general health-related quality of life over a 2-year period.	Sweden	Moderate	Low	Moderate	7
Kemani [54]	2024	Cohort	118 patients—intervention n = 59; conventional care n = 59; average 45.7 years old (SD = 8.3); 53.3% women	Lumbar Spine	To: (1) evaluate the potential long-term effects of the active prehabilitation intervention in comparison to conventional care at the 12- and 24-month follow-up assessments following surgery; and (2) to evaluate changes in all included outcomes over time for both groups	Sweden	Moderate	Moderate	High	7
Knudsen [56]	2023	Case report	83-year-old lady	Degenerative lumbar scoliosis undergoing multilevel spinal fusion	This case report presents the impact of multimodal prehabilitation (MPP) in an octogenarian patient on postoperative clinical and patient-reported outcomes after complex spine surgery.	USA	Moderate	Moderate	High	8
Lam [57]	2022	Qualitative study	Individuals who received (n = 10) and who did not receive (n = 15) prehabilitation before LSS surgery were recruit ted at the 6-month postoperative follow-up (8 females; average age: 67.7 ± 6.7 years)	Lumbar spinal stenosis	To understand patients’ concerns/considerations before LSS surgery, their perspectives toward prehabilitation and experiences after LSS surgery	Hong Kong	High	High	High	5, 6
Lawrence [58]	2023	Retrospective secondary analysis	2203 patients who had elective single-level lumbar fusion spinal surgeries (no exercise; n = 995; Infrequent exercise; n = 245; Regular exercises; n = 963).	Lumbar Fusion Spinal Surgery	To determine whether there was an association between self-reported preoperative exercise and postoperative outcomes after lumbar fusion spinal surgery.	Canada	Moderate	Moderate	High	6, 7
Lindbäck [59]	2018	Randomised controlled trial (PREPARE)	197 patients, mean age 59 y.o (SD 12.5) years, 53% women; 51% had back pain for >2 years; 35% had leg pain for >2 years	Degenerative lumbar spine disorder (includes spinal stenosis, disc herniation or spondylolisthesis)	To study if presurgery physiotherapy improves function, pain, and health in patients with degenerative lumbar spine disorder scheduled for surgery.	Sweden	Moderate	Moderate	High	7, 11

*(continued)*

**Table 1:** Continued

Study	Year	Study design	Participants	Condition	Aim of study	Country	Relevance	Richness	Rigour	CMOCs
Lindbäck [60]	2019	Qualitative study (PREPARE)	18 patients; women (n = 10); median age 65 y.o (range 49–74)	Degenerative lumbar spine disorder	To describe patients’ experiences of how symptoms are explained, and their experiences of the influences on back-related health after pre-surgery physiotherapy	Sweden	High	High	High	1, 7
Lindbäck [61]	2024	Qualitative study (PREPARE)	Patients randomised to pre-surgery physiotherapy in an RCT evaluating the intervention, who had participated in ≥12 sessions, were invited. 18 patients interviewed 0–8 months after pre-surgery physiotherapy, and 16/18 completed a second interview 3–14 months later	Degenerative lumbar spine disorder (includes spinal stenosis, disc herniation or spondylolisthesis)	To describe patients’ pre- and post-surgery experiences after a pre-surgery physiotherapy intervention, and their thoughts about future exercise and self-management.	Sweden	High	High	High	2, 11, 3
Lotzke [62]	2016	Randomised Controlled Trial—Protocol (PREPARE)	110 patients between 18–70 years old with DDD randomised to PREPARE or conventional care	Degenerative Disc Disease (DDD)	To investigate whether PREPARE – a physiotherapeutic prehabilitation program based on a cognitive behavioural approach—will improve functioning after lumbar fusion surgery in patients with DDD compared to conventional care.	Sweden	Moderate	Moderate	High	7
Lotzke [63]	2019	Randomised Controlled Trial	118 patients, Prehab programme (n = 59); mean age 44.8 [SD 8.2]; 33/59 women; Conventional care (n = 59); mean age 46.7 [SD 8.5] 30/59 women	Lumbar degenerative disc disease	To investigate whether a person-centred physical therapy prehabilitation programme based on a cognitive-behavioural approach is more effective than conventional care in reducing disability and improving functioning after lumbar fusion surgery in patients with degenerative disk disease.	Sweden	Moderate	High	High	2, 7, 11
Loughney [64]	2021	Qualitative study	11 male participants mean age60 [SD7]	Cancer (prostate)	To get an insight into men’s perceptions of wellbeing and quality of life following completion of the pre-operative exercise programme	Ireland	Moderate	Low	Moderate	12
Macleod [65]	2018	Feasibility study	22 patients, mean age 67 y.o; 77% male	Cancer (colorectal)	To assess the feasibility of delivering and evaluating a lifestyle programme for patients with colorectal cancer undergoing potentially curative treatments	UK	Moderate	Low	Moderate	2, 3
Mansell [66]	2022	Mediation analysis (PREPARE)	Mediators of interest were exercise self-efficacy; fear of movement, and pain catastrophising	Degenerative lumbar spine disorder (includes spinal stenosis, disc herniation or spondylolisthesis)	To investigate whether early changes in fear of movement (kinesiophobia), self- efficacy and catastrophising were mediators of the relationship be- tween allocation to the prehabilitation intervention and later changes in health outcomes.	Sweden	Moderate	Moderate	High	2,7
Marchand [67]	2019	Randomised pilot/feasibility study	Forty patients (Intervention; n = 20; 9 female average ± SD age 66.7 ± 11.6); control; n = 20; 8 female; average ± SD age; 71.5 ± 7.3)	Lumbar spinal stenosis	To assess the feasibility of conducting a preoperative intervention programme in patients with LSS and to report on the piloting of the proposed intervention.	Canada	High	Moderate	High	5, 9, 3, 14
Marchand [68]	2021	Randomised Controlled Trial	Sixty-eight patients. Mean age intervention group 66.2 ± 9.6; 40% women; Mean age control group 71.6 ± 7.4; 42% women)	Lumbar spinal stenosis	To assess the effectiveness of an active exercise-based prehabilitation program me compared to usual care in patients with LSS	Canada	High	Moderate	High	7, 8
McCarthy [69]	2020	Qualitative study	16 patients (4 female; average ± SD age, 64.3 ± 8.8 years; time since surgery, 9.9 ± 4.4 months) and 10 physical therapists (2 female; average ± SD age, 40.9 ± 6.6 years; time in practice, 17.2 ± 7.7 years)	Lumbar spinal stenosis	To gain the perspectives of patients who underwent LSS) surgery and physical therapists who treat spine-related disorders regarding rehabilitation and other care prior to LSS surgery.	USA	High	High	Moderate	1, 5, 6

*(continued)*

**Table 1:** Continued

Study	Year	Study design	Participants	Condition	Aim of study	Country	Relevance	Richness	Rigour	CMOCs
McCourt [70]	2023	Qualitative study	16 participants (n = 8 intervention group; n = 8 control group; mean age 61 years, 56% male)	Cancer (general)	To explore the experiences of participants who took part in the PERCEPT myeloma pilot trial in order to aid the design of a fully powered RCT.	UK	High	Moderate	Moderate	2, 3, 6, 11
McDonald [71]	2019	Qualitative study	299 participants (65.2% male); median [IQR (range)] age of 68.0 [57.0–76.0 (19.0–91.0)] years	General surgery	To explore patients’ motivation, confidence and priority in relation to changing individual and multiple risk behaviours according to two different temporal frames: changing individual and multiple behaviours for a restricted period to achieve proximal (short-term) peri-operative health benefits; and changing behaviours to achieve distal (long-term) health benefits	UK	Moderate	Low	High	11
Mohamed [72]	2023	Narrative review	128 articles—Included articles focused on frailty and spine surgery, sarcopenia and spine surgery, frailty and prehabilitation in spine surgery, cognitive frailty, nutrition prehabilitation in spine surgery, and ERAS for spine surgery that addressed prehabilitation.	Degenerative spine disease (Cervical, Thoracic and Lumbar Spine)	To provide an overview of frailty assessment in general, as well as the utility and limitations of common frailty assessment tools for spine surgery patients specifically.	N/A	Moderate	Moderate	High	1, 7, 8
Nuevo [73]	2017	Observational study	32 patients, female n = 25 (78%); mean age 72.25 (8.00 SD)	Knee arthroplasty	To demonstrate the effectiveness of the empowerment session in reducing stress associated with knee surgery, establishing a statistically significant correlation between both.	Spain	Moderate	High	High	7
Pellino [74]	1998	Experimental group post-test study	74 patients (experimental group n = 39, mean age 50.43 [SD 21.15], females n = 15; comparison group n = 35) mean age 56.92 (SD 14.67) females n = 22	Orthopaedic surgery	To test a proportion of the proposed model and examine whether patients educated within an empowerment model would report better outcomes than patients educated with a traditional approach	USA	Moderate	High	Moderate	7
Piché [75]	2023	Feasibility study	25 participants, female n = 20(mean age ± SD = 60.2 ± 14.0)	Cancer (general)	To develop a group-based, multimodal, tele-prehabilitation intervention for individuals diagnosed with cancer (iACTIF) and assess its implementation in a ‘real-world’ clinical setting by measuring feasibility, acceptability, fidelity, and preliminary effects	Canada	Moderate	Moderate	High	10, 11
Polen -De [76]	2021	Qualitative study	15 patients;15 female, mean age 64.3	Cancer (ovarian)	To understand and evaluate how patients with advanced ovarian cancer undergoing NACT view exercise and physical activity during treatment	USA	Moderate	Low	High	11
Powell [77]	2023	Qualitative study	Semi-structured interviews—18 participants (engagers (n = 16); (‘non-engagers’(n = 2); Median 68.5 years (range 40s to 80s); 50% female.	Cancer (colorectal, lung or oesophago-gastric cancer)	This study investigated how patients from diverse socio-economic status groups perceived an exemplar prehabilitation and recovery programme, aiming to understand factors impacting acceptability, engagement and referral.	UK	High	High	High	2, 5, 6, 14
Rapp [78]	2021	Case control study	229 patients; patients who participated in preoperative education (n = 113) and those who did not (n = 116).	Elective orthopaedic and neurological spinal surgery	To elucidate the impact of pre-operative education on Hospital Consumer Assessment of Healthcare Providers and Systems (HCAHPS) scores, postoperative pain and length of stay LOS following spinal surgery	USA	Moderate	High	Moderate	5, 7, 12

*(continued)*

**Table 1:** Continued

Study	Year	Study design	Participants	Condition	Aim of study	Country	Relevance	Richness	Rigour	CMOCs
Reyes [79]	2024	Narrative review	Articles that examined spine surgery and preoperative physical therapy in adult patients (>18 years)	Adult spinal surgery	To provide an overview of previously published studies discussing the efficacy of pre-operative rehabilitation program-mes and its role in spinal surgery.	N/A	Moderate	Moderate	Moderate	4, 9
Rolving [80]	2015	Randomised Controlled Trial	90 patients; Intervention group (n = 59, 36 female, age 51.4 (SD ± 9.2), Control group (n = 31, 15 female, age 47.7 (SD ± 8.9)	Degenerative Disc Disease (Lumbar), stenosis or spondylolisthesis grade 1–2	To examine the effect of a pre-operative cognitive behavioural intervention (CBT) for patients undergoing lumbar spinal fusion (LSF) surgery.	Denmark	Moderate	Low	High	7, 9
Rolving [81]	2016	Randomised Controlled trial	90 patients; Intervention group (n = 59, 36 female, age 51.4 (SD ± 9.2), Control group (n = 31, 15 female, age 47.7 (SD ± 8.9)	Degenerative Disc Disease (Lumbar), stenosis or spondylolisthesis grade 1–2	To examine if a preoperative intervention of CBT could influence the early postsurgical outcome following LSF.	Denmark	Moderate	Moderate	High	7, 9
Scarone [82]	2023	Systematic review	13 studies—85% RCTs; 12 Lx fusion surgery (n = 12); Cx fusion surgery (n = 1)	Degenerative or neoplastic spinal disease undergoing a spinal fusion	To summarise the existing evidence about perioperative psychological interventions and to analyse their effect on postoperative pain, disability, and quality of life in adult patients undergoing complex surgery for spinal disorders	N/A	Moderate	Low	High	1
Tang [83]	2020	Qualitative study	Questionnaire (HCPs)—25 responders; 11 Nurse; 7 Doctor; 4 others.	Cancer (prostate)	To utilise Experience-Based Co-Design to identify key design components in a prehabilitation programme for people with prostate cancer and to understand the prostate cancer treatment journey from the perspectives of the patient and health professionals	Australia	Moderate	Low	High	13
Tew [84]	2020	Pilot study	Supervised program me (n = 54) age 69 (42–87) male n = 38 (70%); home-based program me (n = 21) age 68 (51–82) male n = 14 (67%)	General surgery	To implement and evaluate a community-based prehabilitation service for people awaiting elective major surgery: PREP-WELL	UK	High	Moderate	High	6, 9
Thornes [85]	2020	Pilot Study	40 patients; exercise group (n = 26); control group (n = 14).	Lumbar spinal stenosis	To explore the efficacy of a low-impact exercise programme for participants with degenerative spinal stenosis awaiting surgery, aiming to improve physical functioning without deterioration of symptoms.	Norway	High	Moderate	High	5
van der Zanden [86]	2022	Qualitative study	36 participants (n = 16 patients, n = 20 HCP); median age patients 70 y.o (range 62–85 y.o)	Cancer (gynaecological)	To reveal information that can be used for composing a prehabilitation programme tailored to elderly gynaecological oncological patients and is applicable to HCP. We investigated possible content and indications for prehabilitation, and what potential barriers might exist.	Netherlands	High	High	High	1, 2, 6
Voorn [87]	2023	Qualitative study	45 interview, HCP (n = 12), patients (n = 17), informal caregivers (n = 16)	Cancer (lung)	To gain insight into beliefs, facilitators, and barriers of (1) HCP to refer patients to a prehabilitation program, (2) patients with NSCLC to participate in and adhere to a prehabilitation programme, and (3) informal caregivers to support their loved ones in prehabilitation	Netherlands	High	High	High	9, 10, 11, 14

*(continued)*

**Table 1:** Continued

Study	Year	Study design	Participants	Condition	Aim of study	Country	Relevance	Richness	Rigour	CMOCs
Wang [88]	2022	Qualitative study	19 patient interviews (n = 12 female (63%); median age 58 (31-72y.o)	Cancer (colorectal)	To describe experiences and explore preferences for multimodal prehabilitation among colorectal surgery patients.	Canada	High	High	High	3, 5, 6, 8, 11, 12
Wu [89]	2021	Cohort observational study	66 completed questionnaires (34 male; 32 female patients; Age [median (interquartile range)]: 67 (60–73) years	Cancer (general)	To evaluate the feasibility of adapting our preexisting face-to-face programme to a telehealth-delivered home-based format across multiple cancer treatment pathways. The secondary objective was to investigate the effects of our intervention on patient-reported outcomes, with a focus on improving physical function, fatigue, and quality of life upon completion.	UK	Moderate	Moderate	Moderate	9, 12
Wu [90]	2022	Qualitative study	22 patients interviewed (n = 11 female); median age 66 y.o (42–83 y.o)	Cancer (general)	To describe our patients’ perceptions of tele-prehabilitation and capture their capabilities, opportunities, and motivations to participate	UK	High	High	High	2, 3, 9, 12, 13

### Summary of CMOCs

Table 2 summarises the 14 CMOCs created from this review of the prehabilitation literature for people with NC awaiting surgery.

**Table 2:** 14 CMOCs contributing to programme theory

**Communication**
**CMOC 1a Overcoming misconceptions about the nature of prehabilitation** When the prehabilitation programme is clearly distinct from previous conservative treatments and tailored to the patient’s needs and surgical preparation (C), patients interpret it as a purposeful, credible intervention specifically designed to support their surgical recovery (M). This interpretation helps to build trust in the programme’s value, reducing concerns about redundancy or ineffectiveness. As a result, they engage more actively with the programme—participating in exercises and viewing it as a valuable component of their pre-surgical care (O).
**CMOC 1b Avoiding misconceptions about delaying surgery** When prehabilitation is presented as a complementary part of the surgical journey, rather than as a replacement or delay (C), patients reframe their understanding of its purpose—recognising it as an enabler, not a barrier, to surgery (M). This reinterpretation reduces misconceptions and builds confidence that participation will directly support their surgical outcome. As a result, patients demonstrate increased motivation to engage, viewing prehabilitation as a valuable and supportive step toward surgery (O).
**CMOC 5: Clear messaging manages expectations** When HCP consistently emphasise prehabilitation’s valuefor all patients—regardless of health status—highlighting its individualised design and clarifying its rationale and scope (C), patients begin to reconceptualise prehabilitation as personally relevant and purposeful. This fosters a shift in expectations, from seeing it as generic or optional to seeing it as tailored and preparatory (M). Consequently, they engage more actively and confidently, feeling that the programme addresses their unique circumstances and needs (O).
**CMOC 6a: HCP buy-in** When HCP across the multidisciplinary team have ‘bought-in’ and recognise the value of prehabilitation, actively endorsing the programme and implementing a coordinated communication strategy (C), patients perceive a consistent and credible message, which enhances their belief that prehabilitation is trustworthy and worthwhile (M). This strengthened trust motivates greater patient engagement with the prehabilitation process (O).
**CMOC 6b: HCP biomedical-led scepticism** When HCP, particularly those with a strong biomedical orientation, lack a shared understanding or appreciation of the value of prehabilitation (C), patients receive inconsistent and conflicting advice. This confusion prompts patients to question the programme’s legitimacy and reliability, undermining their confidence and trust (M). Consequently, patients are less motivated to engage with the prehabilitation programme (O).
**CMOC 14: Fluctuating waiting times** When a prehabilitation programme is designed flexibly to deliver meaningful content within real-world scheduling constraints and without causing surgical delays (C), patients interpret this flexibility as a sign that the programme respects their time and surgical priorities. This interpretation reduces their anxiety about added burden or delay and increases their confidence in the programme’s relevance (M). Consequently, patients are more motivated to engage with the prehabilitation programme and feel reassured about its place in their surgical journey (O).
Personalisation
**CMOC 2a: Personalised programme** When patients are actively involved in shaping their prehabilitation programme—ensuring it reflects their personal goals, priorities, and perceived needs, with ongoing opportunities to provide feedback and adapt the content accordingly (C), this collaborative process fosters a sense of personal relevance, ownership, and autonomy. This, in turn, strengthens their motivation and confidence that the programme will address what matters most to them (M), leading to greater emotional investment, adherence, and sustained engagement with the programme (O).
**CMOC 2b: Tailored programme** When a prehabilitation programme incorporates comprehensive clinical assessment and profiling to tailor the intervention to the patient’s specific needs across relevant domains (e.g. physical capacity, nutritional status, psychological readiness) (C), patients perceive the programme as personalised and responsive to their unique circumstances. This perception enhances their belief in the programme’s relevance and effectiveness (M), which in turn increases engagement and supports addressing key risk factors to improve surgical readiness (O)
**CMOC 3: Collaborative Goal Setting** When HCP actively engage patients in collaboratively setting realistic and meaningful prehabilitation goals, ensuring these goals align with patients’ priorities and capacities (C), this collaboration fosters a sense of agency, shared ownership, and personal investment in the programme (M). Consequently, patients show increased engagement, motivated by the programme’s relevance and their confidence in achieving these goals (O).
**CMOC 4: Daily Life Prehabilitation: Minimising Burden** When the prehabilitation programme is actively tailored and flexible to fit within patients’ daily routines and time commitments, accommodating their lifestyle and responsibilities (C), patients interpret the programme as a manageable and natural part of their day rather than an additional burden (M). This perception reduces stress and increases adherence, leading to improved overall engagement with the programme (O).
**CMOC 9: Accessible Prehabilitation** When prehabilitation is delivered in accessible, familiar environments such as patients’ homes or local community settings, actively reducing logistical burdens like transportation, parking, and excessive hospital appointments (C), this reduction in practical barriers alleviates stress and enhances patients’ perception of convenience and manageability (M), leading to increased patient engagement with the prehabilitation programme (O).
**CMOC 8: Patient- Centred Nutrition Guidance** When a prehabilitation programme prioritises patient education on the specific benefits of nutrition for surgical preparation and outcomes, providing collaborative, budget-conscious dietary advice that avoids paternalistic weight-loss language (C), patients feel empowered by this knowledge and supportive approach, fostering confidence in their ability to make informed, affordable dietary choices (M), which leads to greater engagement with the nutritional component of the programme (O).
**Support**
**CMOC 7: Patient Education** When LSS prehabilitation includes tailored education on pain catastrophising, fear-avoidance beliefs, and stress reduction techniques aligned with the patient’s condition and surgical expectations (C), this education challenges unhelpful beliefs and equips patients to better understand and manage their thoughts and feelings (M), promoting greater exercise engagement and improved coping (O).

*(continued)*

**Table 2:** Continued

**CMOC 10a: Group Prehabilitation** When a prehabilitation programme for LSS is delivered in a gym or local community centre for patients motivated by group settings and peer support (C), the social environment fosters ongoing encouragement, accountability, and a shared sense of belonging (M), which increases motivation, adherence, and confidence in performing exercises correctly (O).
**CMOC 10b: Private Home-Based Prehabilitation** For individuals sensitive to social pressures, competition, performance anxiety, or those preferring private exercise, a home-based prehabilitation programme offers a safe, non-judgmental environment (C), which reduces social anxiety and embarrassment by fostering feelings of comfort, security, and personal control (M), leading to increased willingness to engage with the prehabilitation programme (O).
**CMOC 11: Support and guidance from a healthcare professional** When patients are personally guided through prehabilitation by the same trusted healthcare professional with specialised knowledge who provides empathetic feedback and tailored advice (C), this ongoing, supportive relationship activates feelings of trust, reassurance, and accountability (M), which motivate patients to engage consistently and adhere to the programme (O).
**CMOC 12: Peer support** When prehabilitation programmes incorporate blended peer support from individuals who have successfully navigated the surgical journey, sharing authentic insights that complement healthcare professional advice (C), this integration of credible lived experience—distinct from family or friend support—activates patient trust, emotional reassurance, and practical motivation (M). Consequently, patients demonstrate increased engagement, driven by the programme’s perceived credibility and unique emotional support (O).
**CMOC 13a: Proactive Telephone Support for Psychological Well-being** When patients on a surgical waiting list receive ongoing, proactive telephone support from HCP, providing updates on their position in the queue and opportunities to voice individual concerns (C), this ongoing communication process fosters a sense of connection, progress, and importance, gradually mitigating feelings of abandonment and vulnerability (M). Consequently, patients experience reduced psychological stress and uncertainty, increased feelings of support, and improved coping abilities during the waiting period (O).
**CMOC 13b: Proactive Telephone Support for Prehabilitation Engagement** When patients on a surgical waiting list receive ongoing, proactive telephone support from HCP, who check on their prehabilitation progress and provide personalised advice (C), patients interpret this personalised engagement as a sign of commitment and care, which reassures them and strengthens their motivation to continue (M). This sense of accountability and encouragement leads to enhanced engagement with prehabilitation, increased motivation, and a stronger sense of responsibility for their progress (O).

### Main findings

**Figure 3:**
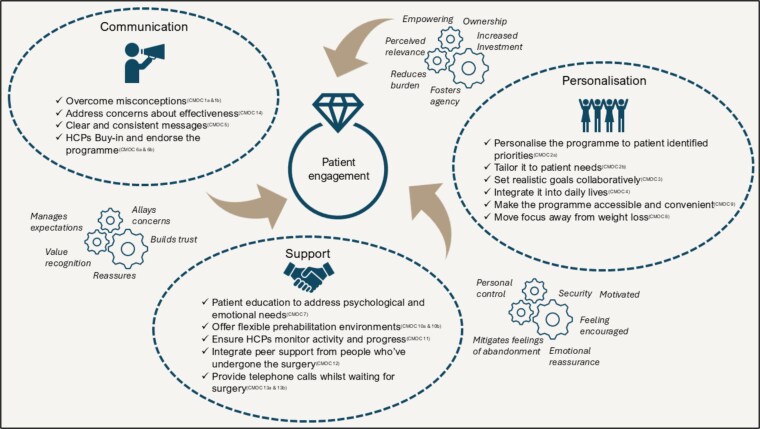
Final programme theory. Thematic divisions are artificial constructs, employed to delineate a cohesive programme theory. Porous boundaries, indicated by dashed circles, acknowledge CMOCs’ potential relevance across multiple themes. Similarly, depicted mechanisms represent salient findings, not exhaustive instances, as some were observed in multiple CMOCs. HCP, healthcare professionals.

Our findings form a programme theory of 14 CMOCs explaining the factors that enhance patient engagement with prehabilitation while awaiting surgery for NC. Structured around three interconnected themes—communication, personalisation and support—these CMOCs highlight key mechanisms and contextual influences on engagement. The final programme theory is illustrated in Figure 3.

#### Theme 1: Communication

Theme one focuses on clear, consistent and credible communication to address patient misconceptions and build trust in prehabilitation. It emphasises the need for healthcare professionals (HCP) to use consistent messages, clearly explain how prehabilitation differs from past treatments, and clarify its purpose to manage expectations and enhance patient engagement.

Patients commonly held misconceptions about prehabilitation, often viewing it as a generic exercise routine [75] already tried during conservative management and found ineffective (CMOC 1a) [30–32, 38, 44, 59, 67, 70, 84]. Some also perceived prehabilitation as an alternative to surgery rather than a preparatory step [67]. Clear, consistent messaging that explicitly differentiated prehabilitation from previous treatments and clarified its role was needed to address this misunderstanding (CMOC 5) [24, 43, 44, 48, 50, 56, 67, 75, 76, 86].

Prehabilitation was best framed as a complementary part of the surgical pathway, rather than a replacement or cause of delay (CMOC 1b) [30–32, 38, 44, 59, 67, 70, 84]. Programmes needed to accommodate real-world scheduling constraints to avoid perceptions of ineffectiveness related to fluctuations in waiting times (CMOC 14) [33, 45, 46, 50, 75, 85]. This flexibility, combined with transparent communication about scheduling, helped reduce anxiety around surgical delays and reinforced the programme’s relevance.

Endorsement of prehabilitation by HCP across the surgical pathway was key to building patient trust (CMOC 6a) [32, 33, 39, 41, 45, 47, 50, 57, 59, 67, 68, 75, 82, 84, 86]. In contrast, inconsistent advice stemming from HCP—especially those with a strong biomedical focus who questioned the value of prehabilitation—undermined patient confidence and engagement (CMOC 6b) [32, 33, 39, 41, 45, 47, 50, 57, 59, 67, 68, 75, 82, 84, 86].

#### Theme 2: Personalisation

Theme two emphasises the importance of tailoring prehabilitation programmes to individual patient needs, preferences and circumstances. It highlights the role of personalised design, collaborative goal-setting and flexible program delivery in fostering patient ownership, relevance and engagement, ensuring that prehabilitation feels like a valuable and bespoke part of their care.

Prehabilitation programmes that were tailored to individual patient needs, preferences and circumstances fostered greater engagement and ownership (CMOC 2a) [14, 27, 28, 30, 39, 40, 44, 46, 48, 50, 58, 60–62, 64, 65, 68, 75, 84, 88]. Patients emphasised the importance of bespoke approaches over generic, ‘one-size-fits-all’ models. Collaborative goal-setting emerged as a key mechanism for engagement, enabling patients to actively participate in shaping their programmes while HCP ensured that goals remained realistic and achievable (CMOC 3) [30, 45, 47, 48, 60, 61, 64, 66, 68, 83, 86, 88, 89].

Risk stratification and patient profiling further enhanced programme relevance by aligning interventions with specific pre-surgical needs, which supported patient trust and perceived value (CMOC 2b). Minimising the burden of prehabilitation was critical to sustained engagement. This included integrating exercises into daily routines (CMOC 4) [46, 59], and addressing practical barriers such as transport and parking through convenient delivery options like home- or community-based programmes (CMOC 9) [25, 28, 31, 40, 50, 51, 66, 77–79, 82, 85, 87, 88].

Personalisation also extended to nutrition advice. While the literature mainly focused on supplementation and weight management, patient feedback emphasised the importance of budget-conscious, tailored nutritional recommendations that respected individual preferences and highlighted broad nutritional benefits rather than focusing solely on weight loss (CMOC 8) [35, 41, 70, 86].

#### Theme 3: Support

This theme centres on the provision of comprehensive support to address the psychological and emotional challenges patients face whilst waiting for surgery. It emphasises the importance of patient education, consistent guidance from trusted HCP, peer support and proactive communication to foster trust, reduce anxiety and enhance engagement in a prehabilitation programme.

Engagement in prehabilitation was strengthened when programmes offered comprehensive psychological and emotional support during the pre-surgical waiting period—a time often marked by stress and uncertainty. Patients described this phase as emotionally challenging, highlighting the importance of targeted support strategies to reduce anxiety and maintain motivation (CMOC 7) [14, 25, 45, 46, 49, 53, 55, 57–61, 65, 70–72, 78, 79, 90]. Patient education that incorporated techniques to address pain catastrophising, fear-avoidance and stress was identified as a key mechanism for alleviating psychological distress and enhancing readiness for surgery.

The delivery environment played a significant role in shaping patients’ experiences of support. Group-based prehabilitation delivered in gyms or community settings was valued by some participants—particularly those who appreciated mutual accountability, camaraderie and a shared sense of progress, which improved confidence and reduced fear (CMOC 10a) [24, 26, 31, 37, 48, 52, 73, 85]. For others, particularly those who experienced social anxiety, preferred privacy, or were deterred by perceived competition, home-based delivery provided a more comfortable and personalised space for engagement (CMOC 10b) [24, 26, 31, 37, 48, 52, 73, 85].

Peer support also contributed significantly to emotional resilience. Structured opportunities for interaction—either online or in person—with individuals who had already undergone surgery provided validation, encouragement and trustworthy advice grounded in lived experience. This form of support was seen by some as more relatable and credible than advice from family or friends [38, 39] and helped reduce feelings of isolation (CMOC 12) [26, 32, 38–40, 47, 48, 52, 63, 76, 86–88].

Proactive telephone contact served as another key form of support. Regular check-ins enabled personalised advice, motivation and progress tracking, while also providing a channel to address individual concerns (CMOC 13b) [28, 32–35, 38, 40, 42, 47, 48, 81, 88]. This contact reassured patients that they had not been forgotten, particularly during surgical delays and offered timely updates on waitlist status, which helped reduce anxiety (CMOC 13a) [28, 32–35, 38, 40, 42, 47, 48, 81, 88].

Support was most effective when it was consistent and came from a trusted HCP who remained involved throughout the prehabilitation journey. A continuous relationship fostered reassurance, accountability and trust—key factors in sustaining engagement through to surgery (CMOC 11) [29–31, 37, 39, 58, 60, 68, 69, 73, 74, 85, 86].

## Discussion

This study builds on findings from a prior realist review on prehabilitation for frail patients [91], where establishing a shared understanding between patients and providers of the intervention’s goals and responsibilities was highlighted as important. In our review, misconceptions—such as viewing prehabilitation as a generic exercise programme or as an alternative to surgery—underscore the need for a clear understanding of what prehabilitation entails and a shared appreciation of its importance. The CMOCs underpinning our programme theory are supported by Lequerica and Kortte’s model of therapeutic engagement [92], which focuses on perceived need, self-efficacy and outcome expectancies that resonate with how patients manage anxiety and respond to prehabilitation while awaiting surgery.

Studies support using risk stratification and profiling to tailor prehabilitation programmes [44, 58]. Tailoring is especially important in resource-limited healthcare settings, ensuring efficient delivery of necessary components and enhancing patient ownership [93]. Generic, non-individualised programmes show poor uptake [44, 65], and patients can struggle with inflexible, hospital-based prehabilitation [50]. A recent study of rehabilitation after LSS surgery found that patients preferred tailored rehabilitation that addressed their individual needs and gave them meaningful activities [94]. However, this emphasis on personalisation makes it challenging to deliver programmes that are both sufficiently flexible to meet individual needs and replicable, scalable and clinically effective.

In response to this challenge, several studies attempted to embed structured frameworks to support both personalisation and fidelity: Edwards *et al*. [44] used the Rehabilitation Treatment Specification System (RTSS) with stakeholder co-design to document and adapt psychoeducational content; Lindbäck *et al*. [58] applied the Treatment-Based Classification (TBC) system with clinician training and checklists to ensure consistency; and Heil *et al*. [50] employed the Ottawa Model for Research Use (OMRU) to map implementation barriers. While these frameworks enhance scalability and reproducibility, they inevitably constrain the depth of co-constructed, individual tailoring—highlighting the ongoing challenge of balancing flexibility with fidelity, scalability and clinical efficacy.

The PREhabilitation, Physical Activity and exeRcisE (PREPARE) trial [62] exemplifies this tension. Although the programme integrated motivational interviewing, patient-led goal setting and tailored therapist-led exercises, it did not sustain significant group differences or conclusively improve disability outcomes for patients awaiting lumbar fusion surgery. The research team noted that delivery became overly reliant on manuals, limiting opportunities for adaptive co-construction with patients. Nevertheless, small to medium effect size trends favoured the intervention—particularly in quality of life and patient-specific functioning—highlighting the potential of personalisation and the importance of adaptability in delivery. The challenges encountered in PREPARE underscore why the programme theory developed in this realist review emphasises individualised co-development, ongoing feedback and flexible implementation to support meaningful engagement and optimise clinical relevance.

Insights from our PAG raised another key tension around tailoring and efficacy: how to design a programme that feels safe and manageable—particularly for older adults with prior negative experiences or health-related anxieties—while still achieving clinically meaningful outcomes. This trade-off was evident in a pilot study by Thornes *et al*. [83], where a low-impact exercise programme was feasible and acceptable for LSS patients awaiting NC surgery but did not improve physical capacity pre-surgery. Yet evidence from other studies suggests that even minimal resistance training—1–2 targeted exercises once or twice a week—can produce measurable strength gains in frail older adults [95]. These contrasting findings underscore the difficulty of balancing patient acceptability, safety and physiological effectiveness when designing prehabilitation programmes.

This tension between clinical effectiveness and individual relevance also extends to nutrition. Surgery for NC is typically performed on older adults (over 65) with LSS; many are likely to be frail [96], with low muscle mass [97] and reduced physical capacity [98]—all factors that increase surgical risk [99]. Accordingly, several studies recommended dietary supplementation and weight-management strategies to optimise outcomes and reduce complications [35, 70]. However, one review found no conclusive evidence that pre-operative weight loss programmes improve post-operative functional outcomes [100]. This aligns with our PAG’s preference to shift dietary advice away from weight reduction and toward broader healthy-eating strategies.

Anxiety and uncertainty during the wait for surgery emerged as key factors influencing how prehabilitation was both experienced by patients and structured by programmes. In response, several studies incorporated cognitive behavioural therapy [46, 65, 79, 101], pain neuroscience education [25, 55, 70], stress reduction techniques [71] and behavioural approaches targeting fear avoidance [53, 102]. These components aimed to reduce surgical fears and address psychological barriers to engagement, such as fear of movement. The frequent inclusion of pain education directly informed our programme theory, underlining its central role in promoting engagement.

However, while educational and psychological strategies target individual cognitive and emotional barriers, many patients also benefit from social and relational support. Peer involvement may enhance motivation, normalise experiences and foster shared understanding—yet its role in prehabilitation for NC patients remains largely unexplored. To date, only one protocol—Baaka *et al*. [26]—included peer sessions for individuals with symptomatic LSS, with results pending. This highlights a gap in the literature and the need for further investigation into the potential benefits and implementation strategies of peer support in this population.

Taken together, these findings highlight the challenge of designing prehabilitation, i.e. both evidence-based and meaningfully tailored to patients with LSS awaiting surgery. Implementing prehabilitation in routine practice may not be feasible until stronger evidence supports its outcomes, particularly given uncertainties around resource allocation and cost responsibilities. The tensions between personalisation and fidelity, safety and efficacy and psychological and physical readiness point to the need for flexible, person-centred approaches grounded in a clear programme theory. While this review offers a provisional programme theory to guide such efforts, further research is needed to identify the core components of an effective prehabilitation programme that can support patient engagement and optimise clinical outcomes.

### Strengths and limitations

A key strength of this realist review was its robust, iterative methodology, guided by expert and PAGs, ensuring the final programme theory was grounded in both clinical and patient experience. Our predominantly white British advisory group (one member of mixed heritage) reflects the demographic most commonly undergoing LSS surgery in the UK, though perspectives of ethnic minority groups may not be fully represented. AI-assisted searches combined with human review improved literature identification efficiency and accuracy, a method increasingly recognised in systematic reviews [103].

A potential limitation is the high proportion of cancer-related studies; however, given limited LSS-specific evidence, drawing on related fields is typical in realist research and was validated through stakeholder consultation. Including diverse surgical populations helps identify context-specific mechanisms and refine interventions.

Due to scarce LSS-specific nutrition literature, CMOC 8 was retained based on advisory input rather than direct evidence. Future research should explore effective prehabilitation components—exercise, dosage, nutrition—and their impact in LSS populations.

## Conclusion

This realist review identified key contexts and mechanisms to enhance prehabilitation engagement for NC patients awaiting surgery. It highlights the need to view prehabilitation as a holistic, individualised preparation—not just a generic intervention. Addressing misconceptions about its purpose is essential to recognising its value. Effective tailoring balances patient empowerment in shaping their programme with ensuring outcomes that improve surgical readiness, recovery and reduce complications. Recognising and addressing psychological barriers like anxiety and fear is crucial to patient engagement and better surgical experiences.

## Supplementary Material

Supplementary_materials_afaf311

## Data Availability

The authors confirm that the data supporting the findings of this study are available within the article and its supplementary materials.

## References

[1] Ravindra VM, Senglaub SS, Rattani A et al. Degenerative lumbar spine disease: estimating global incidence and worldwide volume. Glob Spine J 2018;8:784–94. 10.1177/2192568218770769. PMC6293435 30560029

[2] Deyo RA, Mirza SK, Martin BI et al. Trends, major medical complications, and charges associated with surgery for lumbar spinal stenosis in older adults. JAMA 2010;303:1259–65. 10.1001/jama.2010.338. 20371784 PMC2885954

[3] Deyo RA . Treatment of lumbar spinal stenosis: a balancing act. Spine J 2010;10:625–7. 10.1016/j.spinee.2010.05.006. 20620984

[4] Sang D, Guo J, Meng H et al. Global trends and hotspots of minimally invasive surgery in lumbar spinal stenosis: a bibliometric analysis. J Pain Res 2024;17:117–32. 10.2147/JPR.S440723. 38196967 PMC10775802

[5] The Lancet Rheumatology . Too long to wait: the impact of COVID-19 on elective surgery. Lancet Rheumatol 2021;3:e83. 10.1016/S2665-9913(21)00001-1. 33778775 PMC7987531

[6] Kings Fund . Waiting Times for Elective (Non-urgent) Treatment: referral to Treatment (RTT). London: The King’s Fund, 2021. Available from: https://www.kingsfund.org.uk/insight-and-analysis/data-and-charts/waiting-times-non-urgent-treatment.

[7] Braybrooke J, Ahn H, Gallant A et al. The impact of surgical wait time on patient-based outcomes in posterior lumbar spinal surgery. Eur Spine J 2007;16:1832–9. 10.1007/s00586-007-0452-5. 17701060 PMC2223329

[8] Bailey CS, Gurr KR, Bailey SI et al. Does the wait for lumbar degenerative spinal stenosis surgery have a detrimental effect on patient outcomes? A prospective observational study. Can Med Assoc Open Access J 2016;4:E185–93. 10.9778/cmajo.20150001. PMC4933598 27398362

[9] Jentzsch T, Sundararajan K, Rampersaud YR. The clinical course of symptoms during wait time for lumbar spinal stenosis surgery and its effect on postoperative outcome: a retrospective cohort study. Spine J 2024;24:644–9. 10.1016/j.spinee.2023.11.006. 38008188

[10] Kovacs FM, Urrútia G, Alarcón JD. Surgery versus conservative treatment for symptomatic lumbar spinal stenosis: a systematic review of randomized controlled trials. Spine 2011;36:E1335–51. 10.1097/BRS.0b013e31820c97b1. 21311394

[11] Wynter-Blyth V, Moorthy K. Prehabilitation: preparing patients for surgery. BMJ 2017;358:j3702. 10.1136/bmj.j3702. 28790033

[12] Le Roy , Selvy M, Slim K. The concept of prehabilitation: what the surgeon needs to know? J Visc Surg 2016;153:109–12. 10.1016/j.jviscsurg.2016.01.001. 26851994

[13] Carli F, Scheede-Bergdahl C. Prehabilitation to enhance perioperative care. Anesthesiol Clin 2015;33:17–33. 10.1016/j.anclin.2014.11.002. 25701926

[14] Janssen ERC, Punt IM, Clemens MJ et al. Current Prehabilitation programs do not improve the postoperative outcomes of patients scheduled for lumbar spine surgery: a systematic review with meta-analysis. J Orthop Sports Phys Ther 2021;51:103. 10.2519/jospt.2021.9748. 33356804

[15] Nielsen PR, Jørgensen LD, Dahl B et al. Prehabilitation and early rehabilitation after spinal surgery: randomized clinical trial. Clin Rehabil 2010;24:137–48. 10.1177/0269215509347432. 20103575

[16] Marchand AA, Houle M, O'Shaughnessy J et al. Effectiveness of an exercise-based prehabilitation program for patients awaiting surgery for lumbar spinal stenosis: a randomized clinical trial. Sci Rep 2021;11:11080. 10.1038/s41598-021-90537-4. 34040109 PMC8155114

[17] Hunter R, Gorely T, Beattie M et al. Realist review. Int Rev Sport Exerc Psychol 2022;15:242–65. 10.1080/1750984X.2021.1969674.

[18] Pawson R. Realist synthesis: new protocols for systematic review. In: Fulop N, Allen P, Clarke A, Black N (eds). Evidence-based policy: a handbook of research, practice and debate. London: SAGE, 2006, p. 73–104. 10.4135/9781849209120.n4

[19] The RAMESES Project . Quality standards for realist synthesis (for researchers and peer reviewers). https://www.ramesesproject.org/media/RS_qual_standards_researchers.pdf (21 March 2025, date last accessed).

[20] Wong G, Westhorp G, Manzano A et al. RAMESES II reporting standards for realist evaluations. BMC Med 2016;14:1–18. 10.1186/s12916-016-0643-1. 27342217 PMC4920991

[21] Dada S, Dalkin S, Gilmore B et al. Applying and reporting relevance, richness and rigour in realist evidence appraisals: advancing key concepts in realist reviews. Res Synth Methods 2023;14:504–14. 10.1002/jrsm.1630. 36872619

[22] Crowe M . Crowe critical appraisal tool (CCAT) user guide. Conchra House 2013;10:2–4.

[23] Jagosh J . Retroductive theorizing in Pawson and Tilley’s applied scientific realism. J Crit Realism 2020;19:121–30. 10.1080/14767430.2020.1723301.

[24] Alsaif H, Goodwin PC, Callaghan MJ et al. “Patient and healthcare provider experience and perceptions of a preoperative rehabilitation class for lumbar discectomy: a qualitative study,” (in eng). Musculoskelet Sci Pract 2023;64:102740. 10.1016/j.msksp.2023.102740. 36958123

[25] Arguisuelas MD, Garrigós-Pedrón M, Martínez-Hurtado I et al. The effects of a prehabilitation programme based on therapeutic exercise, back care education, and pain neuroscience education in patients scheduled for lumbar radiculopathy surgery: a study protocol for a randomised controlled trial. PloS One 2024;19:e0303979–9. 10.1371/journal.pone.0303979. 38843271 PMC11156268

[26] Bakaa N, Gross DP, Carlesso LC et al. Presurgical rehabilitation program for patients with symptomatic lumbar spinal stenosis: a pilot randomized controlled trial protocol. Can J Pain-Revue 2022;6:2137009. 10.1080/24740527.2022.2137009.

[27] Barberan-Garcia A, Ubré M, Roca J et al. Personalised prehabilitation in high-risk patients undergoing elective major abdominal surgery: a randomized blinded controlled trial. Ann Surg 2018;267:50–6. 10.1097/SLA.0000000000002293. 28489682

[28] Barnes K, Hladkowicz E, Dorrance K et al. Barriers and facilitators to participation in exercise prehabilitation before cancer surgery for older adults with frailty: a qualitative study. BMC Geriatr 2023;23:356. 10.1186/s12877-023-03990-3. 37280523 PMC10242997

[29] Beck A, Vind Thaysen H, Hasselholt Soegaard C et al. Prehabilitation in cancer care: patients’ ability to prepare for major abdominal surgery. Scand J Caring Sci 2021;35:143–55. 10.1111/scs.12828. 32043644

[30] Beck A, Vind Thaysen H, Hasselholt Soegaard C et al. What matters to you? An investigation of patients’ perspectives on and acceptability of prehabilitation in major cancer surgery. Eur J Cancer Care 2021;30:e13475. 10.1111/ecc.13475 34106493

[31] Beck A, Thaysen HV, Soegaard CH et al. Investigating the experiences, thoughts, and feelings underlying and influencing prehabilitation among cancer patients: a qualitative perspective on the what, when, where, who, and why. Disabil Rehabil 2022;44:202–9. 10.1080/09638288.2020.1762770. 32400218

[32] Bingham SL, Small S, Semple CJ. A qualitative evaluation of a multi-modal cancer prehabilitation programme for colorectal, head and neck and lung cancers patients. PloS One 2023;18:e0277589. 10.1371/journal.pone.0277589. 37788238 PMC10547201

[33] Boukili IE, Flaris AN, Mercier F et al. Prehabilitation before major abdominal surgery: evaluation of the impact of a perioperative clinical pathway, a pilot study. Scand J Surg 2022;111:14574969221083394. 10.1177/14574969221083394. 35437086

[34] Brahmbhatt P, Look Hong NJ, Sriskandarajah A et al. A feasibility randomized controlled trial of Prehabilitation during Neoadjuvant chemotherapy for women with breast cancer: a mixed methods study. Ann Surg Oncol 2024;31:2261–71. 10.1245/s10434-023-14851-z. 38219003

[35] Briguglio M, Wainwright TW. Nutritional and physical Prehabilitation in elective orthopedic surgery: rationale and proposal for implementation. Ther Clin Risk Manag 2022;18:21–30. 10.2147/TCRM.S341953. 35023922 PMC8747789

[36] Bruns ER, Argillander TE, Schuijt HJ et al. Fit4SurgeryTV at-home prehabilitation for frail older patients planned for colorectal cancer surgery: a pilot study. Am J Phys Med Rehabil 2019;98:399–406. 10.1097/PHM.0000000000001108. 30550454

[37] Burke SM, West MA, Grocott MP et al. Exploring the experience of adhering to a prescribed pre-surgical exercise program for patients with advanced rectal cancer: a phenomenological study. Psychol Sport Exerc 2015;16:88–95. 10.1016/j.psychsport.2014.09.005.

[38] Carr T, Teucher U, Casson AG. Waiting for scheduled surgery: a complex patient experience. J Health Psychol 2017;22:290–301. 10.1177/1359105315603464. 26349617

[39] Casanovas-Álvarez A, Sebio-Garcia R, Masià J et al. Experiences of patients with breast cancer participating in a Prehabilitation program: a qualitative study. J Clin Med 2024;13:3732. 10.3390/jcm13133732. 38999298 PMC11242540

[40] Cooper M, Chmelo J, Sinclair RCF et al. Exploring factors influencing uptake and adherence to a home-based prehabilitation physical activity and exercise intervention for patients undergoing chemotherapy before major surgery (ChemoFit): a qualitative study. BMJ Open 2022;12:e062526. 10.1136/bmjopen-2022-062526. PMC9511537 36137639

[41] David Delgado-Lopez P, Rodriguez-Salazar A, Manuel Castilla-Diez J. “Prehabilitation” in degenerative spine surgery: a literature review. Neurocirugia 2019;30:124–32. 10.1016/j.neucir.2018.11.008. 30612856

[42] Deslauriers S, Roy J-S, Bernatsky S et al. The burden of waiting to access pain clinic services: perceptions and experiences of patients with rheumatic conditions. BMC Health Serv Res 2021;21:1–14. 10.1186/s12913-021-06114-y. 33602224 PMC7891805

[43] Eastwood D, Manson N, Bigney E et al. Improving postoperative patient reported benefits and satisfaction following spinal fusion with a single preoperative education session. Spine J 2019;19:840–5. 10.1016/j.spinee.2018.11.010. 30471460

[44] Edwards R, Gibson J, Mungin-Jenkins E et al. A preoperative spinal education intervention for spinal fusion surgery designed using the rehabilitation treatment specification system is safe and could reduce hospital length of stay, normalize expectations, and reduce anxiety a PROSPECTIVE COHORT STUDY. Bone Joint Open 2022;3:135–44. 10.1302/2633-1462.32.BJO-2021-0160.R1. 35139643 PMC8886324

[45] Eubanks JE, Carlesso C, Sundaram M et al. Prehabilitation for spine surgery: a scoping review. PM R 2023;15:1335–50. 10.1002/pmrj.12956. 36730164

[46] Eubanks JE, Cupler ZA, Gliedt JA et al. Preoperative spinal education for lumbar spinal stenosis: a feasibility study. PM&R 2024;16:992–1000. 10.1002/pmrj.13140. 38578142

[47] Francis-Coad J, Edgar D, Bulsara CE et al. Partnering with patients to design a prehabilitation program for optimizing the patient experience through general surgery. Patient Exp J 2021;8:135–47. 10.35680/2372-0247.1544.

[48] Gillis C, Gill M, Gramlich L et al. Patients’ perspectives of prehabilitation as an extension of enhanced recovery after surgery protocols. Can J Surg 2021;64:E578–87. 10.1503/cjs.014420. 34728523 PMC8565881

[49] Gometz A, Maislen D, Youtz C et al. The effectiveness of Prehabilitation (prehab) in both functional and economic outcomes following spinal surgery: a systematic review. Cureus 2018;10:e2675. 10.7759/cureus.2675. 30050730 PMC6059529

[50] Heil TC, Driessen EJM, Argillander TE et al. Implementation of prehabilitation in colorectal cancer surgery: qualitative research on how to strengthen facilitators and overcome barriers. Support Care Cancer 2022;30:7373–86. 35610321 10.1007/s00520-022-07144-w PMC9130002

[51] Heldens A, Bongers BC, de Vos-Geelen et al. Feasibility and preliminary effectiveness of a physical exercise training program during neoadjuvant chemoradiotherapy in individual patients with rectal cancer prior to major elective surgery. Eur J Surg Oncol 2016;42:1322–30. 10.1016/j.ejso.2016.03.021. 27156145

[52] Jandu AK, Nitayamekin A, Stevenson J et al. Post-cancer treatment reflections by patients concerning the provisions and support required for a prehabilitation programme. World J Surg 2023;47:2724–32. 10.1007/s00268-023-07170-7. 37698631 PMC10545643

[53] Kemani MK, Hägg O, Jakobsson M et al. Fear of movement is related to low back disability during a two-year period in patients who have undergone elective lumbar spine surgery. World Neurosurg 2020;137:e416–24. 10.1016/j.wneu.2020.01.218. 32035206

[54] Kemani MK, Hanafi R, Brisby H et al. Long-term follow-up of a person-centered prehabilitation program based on cognitive-behavioral physical therapy for patients scheduled for lumbar fusion. Phys Ther 2024;104:pzae069. 10.1093/ptj/pzae069. PMC11913609 38753831

[55] Knudsen R, Polifka A, Markut KA et al. The impact of multimodal prehabilitation on patient-reported outcomes for a frail octogenarian undergoing multilevel lumbar spinal fusion surgery: a case report. Cureus J Med Sci 2023;15:e46836. 10.7759/cureus.46836. PMC10636751 37954746

[56] Lam AK, Fung OHY, Kwan C et al. The concerns and experiences of patients with lumbar spinal stenosis regarding prehabilitation and recovery after spine surgery: a qualitative study. Arch Rehab Res Clin Transl 2022;4:100227. 10.1016/j.arrct.2022.100227. PMC9761253 36545515

[57] Lawrence DC, Montazeripouragha A, Wai EK et al. Beneficial effects of preoperative exercise on the outcomes of lumbar fusion spinal surgery. Physiother Can 2023;75:22–8. 10.3138/ptc-2021-0030. 37250725 PMC10211389

[58] Lindbäck Y, Tropp H, Enthoven P et al. PREPARE: presurgery physiotherapy for patients with degenerative lumbar spine disorder: a randomized controlled trial. Spine J 2018;18:1347–55. 10.1016/j.spinee.2017.12.009. 29253630

[59] Lindbäck Y, Enthoven P, Öberg B. Patients’ experiences of how symptoms are explained and influences on back-related health after pre-surgery physiotherapy: a qualitative study. Musculoskelet Sci Pract 2019;40:34–9. 10.1016/j.msksp.2019.01.003. 30665046

[60] Lindbäck Y, Carlfjord S. Experiences from pre-surgery physiotherapy and thoughts about future exercise among patients with disc herniation or spinal stenosis: a qualitative study. Musculoskelet Sci Pract 2024;69:102892. 10.1016/j.msksp.2023.102892. 38070465

[61] Lotzke H, Jakobsson M, Brisby H et al. Use of the PREPARE (PREhabilitation, physical activity and exeRcisE) program to improve outcomes after lumbar fusion surgery for severe low back pain: a study protocol of a person-centred randomised controlled trial. BMC Musculoskelet Disord 2016;17:1–13. 10.1186/s12891-016-1203-8. 27538757 PMC4991107

[62] Lotzke H, Brisby H, Gutke A et al. A person-centered prehabilitation program based on cognitive-behavioral physical therapy for patients scheduled for lumbar fusion surgery: a randomized controlled trial. Phys Ther 2019;99:1069–88. 10.1093/ptj/pzz020. 30951604 PMC6665875

[63] Loughney L, McGowan R, O’Malley K et al. Perceptions of wellbeing and quality of life following participation in a community-based pre-operative exercise programme in men with newly diagnosed prostate cancer: a qualitative pilot study. PloS One 2021;16:e0253018. 10.1371/journal.pone.0253018. 34111218 PMC8191992

[64] Macleod M, Steele RJC, O’Carroll RE et al. Feasibility study to assess the delivery of a lifestyle intervention (TreatWELL) for patients with colorectal cancer undergoing potentially curative treatment. BMJ Open 2018;8:e021117. 10.1136/bmjopen-2017-021117. PMC6009630 29880567

[65] Mansell G, den Hollander, Lotzke H et al. A person-centred prehabilitation program based on cognitive behavioural physical therapy for patients scheduled for lumbar fusion surgery: a mediation analysis to assess fear of movement (kinesiophobia), self-efficacy and catastrophizing as mediators of health outcomes. Eur J Pain 2022;26:1790–9. 10.1002/ejp.2004. 35802065 PMC9543490

[66] Marchand A-A, Suitner M, O'Shaughnessy J et al. Feasibility of conducting an active exercise prehabilitation program in patients awaiting spinal stenosis surgery: a randomized pilot study. Sci Rep 2019;9:12257. 10.1038/s41598-019-48736-7. 31439877 PMC6706402

[67] McCarthy AE, Bove AM, Piva S et al. A qualitative study of preparation for lumbar spinal stenosis surgery: perceptions of patients and physical therapists. J Orthop Sports Phys Ther 2020;50:198–205. 10.2519/jospt.2020.8887. 31663813

[68] McCourt O, Fisher A, Land J et al. “What I wanted to do was build myself back up and prepare”: qualitative findings from the PERCEPT trial of prehabilitation during autologous stem cell transplantation in myeloma. BMC Cancer 2023;23:348. 10.1186/s12885-023-10799-1. 37069548 PMC10107576

[69] McDonald S, Yates D, Durrand JW et al. Exploring patient attitudes to behaviour change before surgery to reduce peri-operative risk: preferences for short-vs. long-term behaviour change. Anaesthesia 2019;74:1580–8. 10.1111/anae.14826. 31637700

[70] Mohamed B, Ramachandran R, Rabai F et al. Frailty assessment and Prehabilitation before complex spine surgery in patients with degenerative spine disease: a narrative review. J Neurosurg Anesthesiol 2023;35:19–30. 10.1097/ANA.0000000000000787. 34354024 PMC8816967

[71] Gayoso MN . Do educational and empowerment sessions reduce stress levels before knee arthroplasty. Int J Adv Jt Reconstr 2017;4:19–25.

[72] Pellino T, Tluczek A, Collins M et al. Increasing self-efficacy through empowerment. Orthop Nurs 1998;17:4859. 10.1097/00006416-199807000-00009. 9814337

[73] Piché A, Santa Mina D, Lambert S et al. Assessing real-world implementability of a multimodal group-based tele-prehabilitation program in cancer care: a pragmatic feasibility study. Front Oncol 2023;13:1271812. 10.3389/fonc.2023.1271812. 37965450 PMC10641394

[74] Polen-De C, Langstraat C, Asiedu GB et al. Advanced ovarian cancer patients identify opportunities for prehabilitation: a qualitative study. Gynecol Oncol Rep 2021;36:100731. 10.1016/j.gore.2021.100731. 33718562 PMC7910499

[75] Powell R, Davies A, Rowlinson-Groves K et al. Acceptability of prehabilitation for cancer surgery: a multi-perspective qualitative investigation of patient and ‘clinician’ experiences. BMC Cancer 2023;23:744. 10.1186/s12885-023-10986-0. 37568097 PMC10416438

[76] Rapp A, Sun M, Weissman H et al. Pre-operative patient education does not necessarily reduce length of stay or pain after spinal surgery. Interdiscip Neurosurg Adv Tech Case Manage 2021;24:101044. 10.1016/j.inat.2020.101044.

[77] Reyes JL, Coury JR, Dionne A et al. Preoperative rehabilitation optimization for spinal surgery: a narrative review of assessment, interventions, and feasibility. Spine Deform 2024;12:1261–7. 10.1007/s43390-024-00893-0. 38789728

[78] Rolving N, Nielsen CV, Christensen FB et al. Does a preoperative cognitive-behavioral intervention affect disability, pain behavior, pain, and return to work the first year after lumbar spinal fusion surgery? Spine 2015;40:593–600. 10.1097/BRS.0000000000000843. 25705964

[79] Rolving N, Nielsen CV, Christensen FB et al. Preoperative cognitive-behavioural intervention improves in-hospital mobilisation and analgesic use for lumbar spinal fusion patients. BMC Musculoskelet Disord 2016;17:217. 10.1186/s12891-016-1078-8. 27206497 PMC4875713

[80] Scarone P, Van Santbrink, Koetsier E et al. The effect of perioperative psychological interventions on persistent pain, disability, and quality of life in patients undergoing spinal fusion: a systematic review. Eur Spine J 2023;32:271–88. 10.1007/s00586-022-07426-1. 36427089

[81] Tang CY, Turczyniak M, Sayner A et al. Adopting a collaborative approach in developing a prehabilitation program for patients with prostate cancer utilising experience-based co-design methodology. Support Care Cancer 2020;28:5195–202. 32072326 10.1007/s00520-020-05341-z

[82] Tew GA, Bedford R, Carr E et al. Community-based prehabilitation before elective major surgery: the PREP-WELL quality improvement project. BMJ Open Q 2020;9:e000898. 10.1136/bmjoq-2019-000898. PMC7206908 32213551

[83] Thornes E, Robinson HS, Moosmayer S et al. Let me know if you need anything else and thank you for arranging this. Eur J Physiother 2020;22:97–105. 10.1080/21679169.2018.1554000.

[84] van der Zanden, Van der Zaag-Loonen, Paarlberg KM et al. PREsurgery thoughts–thoughts on prehabilitation in oncologic gynecologic surgery, a qualitative template analysis in older adults and their healthcare professionals. Disabil Rehabil 2022;44:5930–40. 10.1080/09638288.2021.1952319. 34283686

[85] Voorn MJ, Bastiaansen EMW, Schröder CD et al. A qualitative stakeholder analysis of beliefs, facilitators, and barriers for a feasible prehabilitation program before lung cancer surgery. J Cancer Res Clin Oncol 2023;149:15713–26. 10.1007/s00432-023-05298-6. 37668792 PMC10620296

[86] Wang R, Yao C, Hung SH et al. Preparing for colorectal surgery: a qualitative study of experiences and preferences of patients in western Canada. BMC Health Serv Res 2022;22:730. 10.1186/s12913-022-08130-y. 35650598 PMC9161453

[87] Wu F, Rotimi O, Laza-Cagigas R et al. The feasibility and effects of a telehealth-delivered home-based prehabilitation program for cancer patients during the pandemic. Curr Oncol 2021;28:2248–59. 10.3390/curroncol28030207. 34204531 PMC8293185

[88] Wu F, Laza-Cagigas R, Rampal T. Understanding patients’ experiences and perspectives of tele-prehabilitation: a qualitative study to inform service design and delivery. Clin Pract 2022;12:640–52. 10.3390/clinpract12040067. 36005070 PMC9406597

[89] Bruns ER, van Rooijen SJ, Argillander TE et al. Improving outcomes in oncological colorectal surgery by prehabilitation. Am J Phys Med Rehabil 2019;98:231–8. 10.1097/PHM.0000000000001025. 30153125

[90] Baldoman D, Vandenbrink R. Physical therapy challenges in head and neck cancer. Cancer Treat Res 2018;174:209–23. 10.1007/978-3-319-65421-8_12. 29435844

[91] Sontag AF, Kiselev J, Schaller SJ et al. Facilitators and barriers to the implementation of prehabilitation for frail patients into routine health care: a realist review. BMC Health Serv Res 2024;24:192. 38350947 10.1186/s12913-024-10665-1 PMC10863196

[92] Lequerica AH, Kortte K. Therapeutic engagement: a proposed model of engagement in medical rehabilitation. Am J Phys Med Rehabil 2010;89:415–22. 20407308 10.1097/PHM.0b013e3181d8ceb2

[93] Riedel B, Ismail H, Denehy L et al. Transforming surgical waiting lists into preparation opportunities: leveraging multimodal Prehabilitation to optimise surgical outcomes. ANZ J Surg 2025;95:12–16. 10.1111/ans.19307. 39540561

[94] McIlroy S, Brighton L, Weinman J et al. Experiences of recovery and rehabilitation from surgery to treat neurogenic claudication. A qualitative study. Disabil Rehabil 2024;47:4234–43. 10.1080/09638288.2024.2442531. 39714172

[95] Byrne C, Faure C, Keene DJ et al. Ageing, muscle power and physical function: a systematic review and implications for pragmatic training interventions. Sports Med 2016;46:1311–32. 10.1007/s40279-016-0489-x. 26893098

[96] Kim H-J, Park S, Park SH et al. The prevalence and impact of frailty in patients with symptomatic lumbar spinal stenosis. Eur Spine J 2019;28:46–54. 10.1007/s00586-018-5710-1. 30173327

[97] Park S, Kim HJ, Ko BG et al. The prevalence and impact of sarcopenia on degenerative lumbar spinal stenosis. Bone Joint J 2016;98:1093–8. 27482023 10.1302/0301-620X.98B8.37623

[98] Tomkins-Lane C, Melloh M, Lurie J et al. ISSLS prize winner: consensus on the clinical diagnosis of lumbar spinal stenosis: results of an international Delphi study. Spine 2016;41:1239–46. 10.1097/BRS.0000000000001476. 26839989 PMC4966995

[99] Briguglio M . Nutritional orthopedics and space nutrition as two sides of the same coin: a scoping review. Nutrients 2021;13:1–28. 10.3390/nu13020483. PMC7912880 33535596

[100] Delgado-López PD, Rodríguez-Salazar A, Castilla-Díez JM. “Prehabilitation” in degenerative spine surgery: a literature review. Neurocirugía 2019;30:124–32. 10.1016/j.neucie.2018.11.002. 30612856

[101] Rolving N, Sogaard R, Nielsen CV et al. Preoperative cognitive-behavioral patient education versus standard care for lumbar spinal fusion patients: economic evaluation alongside a randomized controlled trial. Spine 2016;41:18–25. 10.1097/BRS.0000000000001254. 26536443

[102] Lindbäck Y, Tropp H, Enthoven P et al. “PREPARE: pre-surgery physiotherapy for patients with degenerative lumbar spine disorder: a randomized controlled trial protocol,” (in eng). BMC Musculoskelet Disord 2016;17:270. 10.1186/s12891-016-1126-4. 27400960 PMC4940916

[103] Roth S, Wermer-Colan A. Machine learning methods for systematic reviews: a rapid scoping review. Delaware J Public Health 2023;9:40. 10.32481/djph.2023.11.008 PMC10759980 38173960

