# Supplementary material for: Prehabilitation for lumbar spinal stenosis: understanding mechanisms and contexts for enhanced engagement—a realist review
Source: Age Ageing. 2025 Oct 24;54(10):afaf311. doi: 10.1093/ageing/afaf311 (PMC12551379; doi:10.1093/ageing/afaf311)
Supplement: Supplementary_materials_afaf311 [file supplementary_materials_afaf311.zip › Supplementary_materials_afaf311_File003.docx]

**Appendix 2: Patient and Expert Advisory Group**

**Patient Advisory Group (PAG)**

As part of this realist review, a Patient Advisory Group (PAG) was created to contribute lived experience insights, ensure relevance of the programme theory, and assist in refining Context-Mechanism-Outcome Configurations (CMOCs). Participants were recruited through an advertisement emailed by the PenARC Patient and Public Involvement and Engagement Team to their list of public and patient members. The group participated in two stakeholder consultations held on **July 4th and September 12th, 2024**.

**Composition of the PAG**

The group consisted of **seven older adults** (five female, two male), all of whom had undergone spinal surgery for neurogenic claudication (NC) related to lumbar spinal stenosis (LSS).

| **Participant** | **Gender** | **Age Range** | **Ethnicity** | **Location in England** |
| --- | --- | --- | --- | --- |
| 1 | Female | 60–70 | White British | Kent |
| 2 | Male | 80–90 | White British | Kent |
| 3 | Female | 70–80 | White Other | Yorkshire |
| 4 | Female | 50–60 | White British | London |
| 5 | Male | 70–80 | White British | Kent |
| 6 | Female | 80–90 | White British | London |
| 7 | Female | 60–70 | Mixed | Nottingham |

The involvement of this diverse group ensured the incorporation of valuable experiential knowledge to inform the review findings.

**Expert Advisory Group**

The Expert Advisory Group was gathered to provide specialist clinical, academic, and physiological insights relevant to the development and refinement of the programme theory. Members were recruited through existing professional networks and relationships. Members participated in two stakeholder consultations held on **April 24th and September 18th, 2024**.

**Composition of the Expert Advisory Group**

- **Consultant Physician in Ortho-Geriatric Medicine**
- **Consultant Physiotherapist** specialising in spinal surgery
- **Consultant Physiotherapist** specialising in musculoskeletal conditions
- **Academic Physiotherapist** with expertise in lumbar spinal stenosis and rehabilitation in older adults
- **Professor of Translational Physiology**
- **Clinical Exercise Physiologist**

These experts contributed domain-specific knowledge to enhance the rigour and clinical relevance of the Context-Mechanism-Outcome Configurations (CMOCs) developed in the review.
