# Supplementary material for: Prehabilitation for lumbar spinal stenosis: understanding mechanisms and contexts for enhanced engagement—a realist review
Source: Age Ageing. 2025 Oct 24;54(10):afaf311. doi: 10.1093/ageing/afaf311 (PMC12551379; doi:10.1093/ageing/afaf311)
Supplement: Supplementary_materials_afaf311 [file supplementary_materials_afaf311.zip › Supplementary_materials_afaf311_File004.docx]

**Appendix 3: Literature search 1 search log**

| **Source** | **Date searched** | **Search strategy** | **Hits (or records obtained from searches)** | **Comments** |
| --- | --- | --- | --- | --- |
| PubMed | 10/06/2024 | Search Actions Details Query Results Time  #14 Search: #12 NOT #5 Filters: from 2011 - 2024 Sort by: Most Recent  #15 Search: #1 AND #14 Sort by: Most Recent  20 05:25:37  #13 Search: #12 NOT #5 Sort by: Most Recent  137 05:23:45  #12 Search: "preoperative rehabilitation" Sort by: Most Recent  166 05:22:43  #11 Search: preoperative rehabilitation Sort by: Most Recent  15,105 05:22:25#8 Search: #5 NOT #7 Sort by: Most Recent  #7 Search: #5 AND #6 Sort by: Most Recent  #6 Search: prehabilitation[tiab] Sort by: Most Recent  1,944 04:56:29  #5 Search: #1 AND #2 Filters: English, from 2011 - 2024 Sort by: Most Recent  567 04:54:03  #2 Search: prehabilitation Sort by: Most Recent  6,204 04:52:18  #4 Search: #1 AND #2 Filters: from 2011 - 2024 Sort by: Most Recent  634 04:51:15  #3 Search: #1 AND #2 Sort by: Most Recent  799 04:50:29  #1 Search: Orthopedic Procedures Sort by: Most Recent  382,639 04:48:19 | **113 05:26:28**  **460 05:02:31**  **107 05:00:29** | Preoperative rehabilitation (excluding below)  Remainder – Moderate relevance prehabilitation  High relevance - prehabilitation |
| **CINAHL** | **10/06/2024** | **Search ID# Search Terms Search Options**  S8 S3 OR S5  **Limiters** - Publication Date: 20110101-20241231  **Narrow by Language:**- english  **Search modes** - Boolean/Phrase [**View Results**](javascript:__doPostBack('ctl00$ctl00$FindField$FindField$historyControl$HistoryRepeater$ctl00$linkResults','')) (118) [**View Details**](javascript:showShDetails(%22ctl00_ctl00_FindField_FindField_historyControl_ctrlPopup%22,%20%22S8%22,%20true);)  S7 S3 OR S5  **Narrow by Language:**- English **Search modes** - Boolean/Phrase [**View Results**](javascript:__doPostBack('ctl00$ctl00$FindField$FindField$historyControl$HistoryRepeater$ctl01$linkResults','')) (130) [**View Details**](javascript:showShDetails(%22ctl00_ctl00_FindField_FindField_historyControl_ctrlPopup%22,%20%22S7%22,%20true);)  **S6** S3 OR S5 [**View Results**](javascript:__doPostBack('ctl00$ctl00$FindField$FindField$historyControl$HistoryRepeater$ctl00$linkResults','')) (135) [**View Details**](javascript:showShDetails(%22ctl00_ctl00_FindField_FindField_historyControl_ctrlPopup%22,%20%22S6%22,%20true);)S5 ((MH "Orthopedic Surgery+")) AND (S1 AND S4) [**View Results**](javascript:__doPostBack('ctl00$ctl00$FindField$FindField$historyControl$HistoryRepeater$ctl01$linkResults','')) (131) [**View Details**](javascript:showShDetails(%22ctl00_ctl00_FindField_FindField_historyControl_ctrlPopup%22,%20%22S5%22,%20true);)  **S4 (MH "Orthopedic Surgery+") View Results (135,842) View Details**  **S3 ((MH "Spinal Diseases+")) AND (S1 AND S2) View Results (7) View Details**  **S2 (MH "Spinal Diseases+") View Results (40,199) View Details**  **S1 prehabilitation or prehab or pre-operative rehabilitation or peri-operative rehabilitation View Results (975) View Details** | **118 records** | **Limited to English 2011-2024** |
| **Scopus** | **10/06/2024** | **( ALL ( prehabilitation OR "preoperative rehabilitation" OR "pre-operative rehabilitation" ) AND TITLE-ABS-KEY ( lumbar OR spine OR spinal OR orthoped* OR orthopaed* ) ) AND PUBYEAR > 2010 AND PUBYEAR < 2025 AND ( LIMIT-TO ( LANGUAGE , "English" ) ) AND ( LIMIT-TO ( SUBJAREA , "MEDI" ) OR LIMIT-TO ( SUBJAREA , "HEAL" ) OR LIMIT-TO ( SUBJAREA , "NURS" ) ) AND ( LIMIT-TO ( EXACTKEYWORD , "Human" ) )** | **612 documents** | **NB. No Subject Indexing of Orthop(a)edic Surgery** |
| **Web of Science** | **10/06/2024** | **prehabilitation OR "Preoperative rehabilitation" OR "pre operative rehabilitation" (Topic)**  **Refined By:NOT Database: Preprint Citation Index.**  **Publication Years: 2024 or 2023 or 2022 or 2021 or 2020 or 2019 or 2018 or 2017 or 2016 or 2015 or 2014 or 2013 or 2012 or 2011.**  **Search within topic: Orthopaed* Or Orthoped* Or Lumbar Or Spinal Or Spine.**  **Languages: English** | **238 results from All Databases** |  |
