# Supplementary material for: Prehabilitation for lumbar spinal stenosis: understanding mechanisms and contexts for enhanced engagement—a realist review
Source: Age Ageing. 2025 Oct 24;54(10):afaf311. doi: 10.1093/ageing/afaf311 (PMC12551379; doi:10.1093/ageing/afaf311)
Supplement: Supplementary_materials_afaf311 [file supplementary_materials_afaf311.zip › Supplementary_materials_afaf311_File005.docx]

**Appendix 4: Scite literature results for CMOC 3**

Scite: prehabilitation AND (individualised or Tailored or individualized OR tailor OR individual)

“…Recent evidence supports prehabilitation as an effective strategy towards high-value surgical care. Besides improving preoperative and postoperative functional status [[24,](https://scite.ai/reports/10.1007/s00464-012-2560-5)[25]](https://scite.ai/reports/10.1001/jamasurg.2018.1645), prehabilitation can reduce postoperative complications and hence treatment cost [[26,](https://scite.ai/reports/10.1002/bjs.7102)[27,](https://scite.ai/reports/10.1016/j.surg.2016.05.014)[28]](https://scite.ai/reports/10.1016/j.bja.2019.05.032).…”

Section: Adherence To Prehabilitationmentioning

“…It was suggested that functional outcomes were so much more important to patients [[1]](https://scite.ai/reports/10.1111/j.1532-5415.2010.03113.x). Our group went on to focus on driving consistency in optimising functional outcomes for older patients after major surgery [[2,](https://scite.ai/reports/10.1007/s00268-011-1112-9)[3]](https://scite.ai/reports/10.1111/jgs.12584). During this process, attention to frailty was one of the main foci and we went on to achieve good functional results and surgical outcome improvements through prehabilitation for the frail older surgical patient [[4,](https://scite.ai/reports/10.1111/codi.13166)[5]](https://scite.ai/reports/10.12809/ajgg-2018-304-oa).…”

Section: Introductionmentioning

“…The rationale of care bundles is the delivery of elements of care that improve outcomes when combined, as exemplified by bundles for sepsis, ventilator care, and prevention of surgical site infections. The concept is also applicable to larger systems, as seen in the successful implementation of the Emergency Laparotomy Pathway Quality Improvement Care bundle in reducing mortality [[11]](https://scite.ai/reports/10.1002/bjs.9658). The concept of multimodal prehabilitation as a bundle has been systematically reviewed, although further large prospective trials are needed [[12]](https://scite.ai/reports/10.1097/dcr.0000000000000987).…”

Section: Prehabilitation As a Multimodal Bundle

“…This suggests that colorectal cancer patients may reap the most benefit from prehabilitation within an optimal time frame to allow for postoperative recovery and avoid delays in adjuvant chemotherapy. Similarly, for patients undergoing lobectomy for lung cancer, recent evidence [[35]](https://scite.ai/reports/10.1510/icvts.2010.264507) has shown that a preoperative structured exercise programme of four to six weeks delivers improvements in aerobic capacity comparable to traditional postoperative pulmonary rehabilitation lasting 12 to 15 weeks. Preoperative prehabilitation programmes are now advocated to improve exercise capacity in patients undergoing other major thoracic surgeries.…”

Section: Adherence To Prehabilitation

“…Porter's methodology for value measurement represents outcomes divided by costs incurred [[43]](https://scite.ai/reports/10.1097/sla.0b013e31818a43af). Expanding upon the concepts of outcome and cost as applied to the context of prehabilitation, we propose that assessment of the true value of prehabilitation encompasses these four dimensions:…”

Section: Value Analysis In Prehabilitation

See 3 more Smart Citations

[Finding value with prehabilitation in older persons receiving surgery](https://scite.ai/reports/finding-value-with-prehabilitation-in-mOP8PVmy)

[Priscilla Ng](https://scite.ai/authors/priscilla-ng-lyrblb)^1^,

[Jin Keat Daniel Lee](https://scite.ai/authors/jin-keat-daniel-lee-WNY6PJ)^2^,

[Kok‐Yang Tan](https://scite.ai/authors/kok-yang-tan-685YJg)^3^

2021

[*Current Opinion in Supportive and Palliative Care*](https://scite.ai/journals/1751-4258)

“…Importantly, some of the aforementioned studies also include qualitative process evaluations (Macleod et al, [2018](https://scite.ai/reports/10.1136/bmjopen-2017-021117); Brahmbhatt et al, [2020](https://scite.ai/reports/10.3389/fonc.2020.571091); McCourt et al, [2020](https://scite.ai/reports/10.1136/bmjopen-2019-033176)). Brahmbhatt et al ( [2020](https://scite.ai/reports/10.3389/fonc.2020.571091)) present findings from interviews with participants who had participated in a home-based exercise prehabilitation program prior to breast cancer surgery.…”

Section: Recent and Ongoing Prehabilitation Trialsmentioning

confidence: 99%

“…We encourage those designing new trials to include qualitative process evaluations by following published guidance (Moore et al, [2015](https://scite.ai/reports/10.1136/bmj.h1258)). Not only does this allow exploration of patient experience, it can shed light on what worked for whom and in what context; vital data to support advancement and implementation of prehabilitation trials and services.…”

Section: Recent and Ongoing Prehabilitation Trialsmentioning

confidence: 99%

“…Furthermore, a psychological determinant of behavior change that has received considerable attention is self-efficacy. Defined as “ the belief in one's capabilities to organize and execute the courses of action required to produce given attainments,” self-efficacy has been established as one of the most consistent predictors of adoption and maintenance of physical activity behavior (van Stralen et al, [2009](https://scite.ai/reports/10.1080/17437190903229462)). Studies exploring patient perceptions and experience of prehabilitation programs describe high motivation to engage but confidence to do so as low, hindering engagement (McDonald et al, [2019](https://scite.ai/reports/10.1111/anae.14826); Beck et al, [2020](https://scite.ai/reports/10.1080/09638288.2020.1762770)).…”

Section: The Role Of Theories and Framework Of Behavior Changementioning

confidence: 99%

“…and to foster patients' engagement for healthy lifestyles” p. 4 (Barberan-Garcia et al, [2020](https://scite.ai/reports/10.1186/s12913-020-05078-9)). Furthermore, in an ongoing trial described by McCourt et al ( [2020](https://scite.ai/reports/10.1136/bmjopen-2019-033176)) the role of behavioral science is explicitly described. This study will investigate the feasibility of exercise-based prehabilitation prior to stem-cell transplantations in myeloma patients.…”

Section: Recent and Ongoing Prehabilitation Trialsmentioning

“…This could include for example, allowing choices where possible, setting graded tasks, and in the context of exercise, prescribing affect-regulated exercise [i.e., an intensity that feels good (Parfitt et al, [2012](https://scite.ai/reports/10.1016/j.jsams.2012.01.005))]. These strategies should enhance enjoyment of the program and in doing so increase the likelihood of on-going behavior change (Teixeira et al, [2012](https://scite.ai/reports/10.1186/1479-5868-9-78)). Incorporating strategies to promote habit formation could also help to achieve longer-term outcomes (Gardner et al, [2012](https://scite.ai/reports/10.3399/bjgp12x659466)).…”

Section: The Role Of Theories and Framework Of Behavior Changementioning

confidence: 99%

See fewer Smart Citations

[The Role of Behavioral Science in Personalized Multimodal Prehabilitation in Cancer](https://scite.ai/reports/the-role-of-behavioral-science-J1KLpYzE)

[Chloe Grimmett](https://scite.ai/authors/chloe-grimmett-RVn9a9)^1^,

[Katherine Bradbury](https://scite.ai/authors/katherine-bradbury-zRaEmx)^2^,

[Susanne Oksbjerg Dalton](https://scite.ai/authors/susanne-oksbjerg-dalton-XegRxy)^3^

et al. 2021

[*Front. Psychol.*](https://scite.ai/journals/1664-1078)

“…This study group has previously published and documented in an RCT study that multimodal prehabilitation before RC was feasible and led to a positive and significant change in patients' fitness and functional status compared with the standard protocols after surgery. Moreover, specific health-related quality-of-life items such as respiratory and abdominal function, as defined by the European Organization for Research and Treatment of Cancer (EORTC), were improved from a short-term perspective [[3,](https://scite.ai/reports/10.1016/j.euf.2019.05.016)[[11]](https://scite.ai/reports/10.1007/s00520-016-3140-3)[[12]](https://scite.ai/reports/10.7257/1053-816x.2019.39.6.303)[[13]](https://scite.ai/reports/10.2147/jmdh.s62172). Currently, only a short-term follow-up of eight weeks has been reported after multimodal prehabilitation.…”

Section: Introductionmentioning

confidence: 99%

“…Enhanced mobilization was defined by everyday goals concerning walking distance and hours out of bed. Patients in the standard group did not receive prehabilitation intervention but only the standardized national recommendation regarding physical activity and nutritional care when in line with international recommendations [[15,](https://scite.ai/reports/10.1249/mss.0000000000002117)[16]](https://scite.ai/reports/10.1016/j.clnu.2017.02.013). Moreover, patients were postoperatively mobilized according to the standardized ERAS protocol.…”

Section: The Physical Componentmentioning

confidence: 99%

“…The study included patients scheduled for RC because of localized MIBC or high-risk NMIBC during the study period, leaving 50 patients in the intervention group (nI = 50) and 57 patients in the standard group (ns = 57). Further information on the inclusion, randomization process, and Consort flowchart has been previously published [[14]](https://scite.ai/reports/10.3109/21681805.2014.967810) (Table 1).…”

Section: Methodsmentioning

confidence: 99%

“…Despite the Enhanced Recovery after Surgery (ERAS) protocols, including the significant improvement in anesthetic protocols, minimal-invasive surgical techniques, and optimized postoperative care protocols, the rate of postoperative complications remains high. Therefore, prehabilitation has become an emergent clinical focus area with the specific aim to reduce modifiable risk factors before surgery and optimize functional status [[3,](https://scite.ai/reports/10.1016/j.euf.2019.05.016)4]. In an ERAS context, multimodal prehabilitation is the process of enhancing physiological, nutritional, and psychological resilience to increase patients' functional capacity before major cancer surgery [[5,](https://scite.ai/reports/10.5489/cuaj.2025)[6]](https://scite.ai/reports/10.1097/aln.0000000000000393) and aims to empower the patient to withstand the pending stress of major surgery, improve the rate of postoperative recovery, and ultimately, through behavioral change, improve long-term health outcomes and survivorship [[7,](https://scite.ai/reports/10.1097/eja.0000000000001167)[8]](https://scite.ai/reports/10.1177/0269215518815531).…”

Section: Introductionmentioning

“…Therefore, prehabilitation has become an emergent clinical focus area with the specific aim to reduce modifiable risk factors before surgery and optimize functional status [[3,](https://scite.ai/reports/10.1016/j.euf.2019.05.016)4]. In an ERAS context, multimodal prehabilitation is the process of enhancing physiological, nutritional, and psychological resilience to increase patients' functional capacity before major cancer surgery [[5,](https://scite.ai/reports/10.5489/cuaj.2025)[6]](https://scite.ai/reports/10.1097/aln.0000000000000393) and aims to empower the patient to withstand the pending stress of major surgery, improve the rate of postoperative recovery, and ultimately, through behavioral change, improve long-term health outcomes and survivorship [[7,](https://scite.ai/reports/10.1097/eja.0000000000001167)[8]](https://scite.ai/reports/10.1177/0269215518815531).…”

Section: Introductionmentioning

See 3 more Smart Citations

[One-Year Follow-Up after Multimodal Prehabilitation Interventions in Radical Cystectomy](https://scite.ai/reports/one-year-follow-up-after-multimodal-prehabilitation-1Z8kmw5y)

Bente Thoft Jensen

2023

[*Cancers*](https://scite.ai/journals/2072-6694)

“…Another important and novel finding from this study was that nearly 70% of patients reported stress reduction as one of the goals for this multimodal prehabilitation programme (only six indicated this as ‘not relevant’), and nearly 60% worked on stress reduction at least once a week. Other prehabilitation studies involving psychological support have reported adherence rates of 96% for a supportive attention intervention [[41](https://scite.ai/reports/10.1200/jco.2007.16.0036)] and 86% for an audio CD intervention [[42](https://scite.ai/reports/10.1002/pon.3034)]. In both Garssen et al.…”

Section: Discussionmentioning

confidence: 99%

“…Surgery, however, puts significant stress on a body that has a multifactorial pathophysiology, leading to multiple postoperative complications and permanent reduction in functional capacity and quality of life (QoL) [[4, 5](https://scite.ai/reports/undefined)]. Despite minimally invasive surgical techniques and perioperative Enhanced Recovery After Surgery (ERAS) programmes, complications after colorectal surgery still occur in roughly one‐third of patients; specifically, one‐fifth of patients experience major complications and one‐sixth experience minor complications [[6](https://scite.ai/reports/10.1016/j.ejso.2015.03.236)]. Postoperative ileus is one minor complication that affects 10%–30% of colorectal patients and causes significant patient morbidity and reduced QoL [[7](https://scite.ai/reports/10.1016/j.jviscsurg.2016.08.010)].…”

Section: Introductionmentioning

confidence: 99%

“…The components of PREP were chosen based on validated resources from the Strong for Surgery toolkit that emphasizes smoking cessation, being active, nutrition and social support [33]. The stress management aspect of ‘healthy coping’ was especially emphasized in PREP, as our previous work found a need and strong preference for mental/emotional health support [[23](https://scite.ai/reports/10.1186/s12913-022-08130-y)].…”

Section: Methodsmentioning

“…The PAG was formed from two formal patient partners who agreed to be named on the grant and three additional patient partners who responded favourably to our initial invitations to join the patient‐oriented project. The PAG consisted of patient partners who had undergone colorectal surgical procedures in the past and had been invited from the participant list of our previous focus group research on experiences of and preferences for prehabilitation [[23](https://scite.ai/reports/10.1186/s12913-022-08130-y)].…”

Section: Methodsmentioning

“…In British Columbia, the average preoperative waiting times for 90% of cases were completed within 21 weeks for colostomy/ileostomy, 21 weeks for laparoscopy and 30 weeks for rectal surgery between June 2023 and August 2023 [11]. There is a growing body of research focused on earlier intervention in this preoperative phase, called prehabilitation, that may serve as a complementary strategy to mitigate the risk of postsurgical complications and reduce the healthcare costs of colorectal surgery by optimizing patients' preoperative physiological reserves [[12]](https://scite.ai/reports/10.1097/nor.0000000000000264)[[13]](https://scite.ai/reports/10.1016/j.pmr.2016.09.002)[[14]](https://scite.ai/reports/10.1007/s00540-017-2329-z)[[15]](https://scite.ai/reports/10.1093/bja/78.5.606)[[16]](https://scite.ai/reports/10.1139/apnm-2015-0084)[[17]](https://scite.ai/reports/10.1097/aco.0b013e32834ef903). Most studies on prehabilitation interventions have typically focused on exercise as the main component, sometimes with nutritional and/or psychological support [[18,](https://scite.ai/reports/10.1016/j.ijsu.2021.106079)[19]](https://scite.ai/reports/10.3389/fresc.2021.650835).…”

Section: Introductionmentioning

See 3 more Smart Citations

[Preparing for colorectal surgery: a feasibility study of a novel web‐based multimodal prehabilitation programme in Western Canada](https://scite.ai/reports/preparing-for-colorectal-surgery-a-mORb8Z2g)

Nathanael Ip

2024

[*Colorectal Disease*](https://scite.ai/journals/1462-8910)

“…For a cohort of women in Denmark, prehabilitation was considered acceptable if it fitted in with their everyday lives and allowed them to carry out other tasks which helped them ‘prepare’ for surgery, such as meal preparation, laundry, gardening [ [40](https://scite.ai/reports/10.1111/ecc.13475), [41](https://scite.ai/reports/10.1080/09638288.2020.1762770)]. Following the recommendations provided, women were ready to accept prehabilitation as being beneficial for health and wellbeing, but spending time with loved ones, funeral planning and finances were considered equally as important by some.…”

Section: Resultsmentioning

“…Gynaecological cancers consist of vulvar, cervical, vaginal, endometrial, and ovarian tumours. The latter in particular are associated with increased mortality and morbidity, often due to late and advanced presentation [ [6](https://scite.ai/reports/10.1158/1055-9965.epi-16-0520), [7](https://scite.ai/reports/10.1136/ijgc-2021-002951)]. Women with endometrial cancer have better survival overall, but over 50% are obese and therefore at risk of cardiovascular disease and other co-morbidities [ [8](https://scite.ai/reports/10.1093/annonc/mdw310)].…”

Section: Introductionmentioning

confidence: 99%

“…This review follows the Joanna Briggs Institute (JBI) guidelines for scoping reviews [ [18](https://scite.ai/reports/10.1097/xeb.0000000000000277)], Preferred Reporting Items for Systematic reviews and Meta-Analyses extension for Scoping Reviews (PRISMA-ScR) checklist [ [14](https://scite.ai/reports/10.7326/m18-0850)]. The review protocol has been published in an open access forum [ 19 ].…”

Section: Methodsmentioning

confidence: 99%

“…Step 1 Define the review questions Given the complexity of prehabilitation as an intervention, it is important not only to understand what has worked or is perceived to work based on measured or predicted outcomes, but also the mechanisms and the context which may operate as facilitators and barriers, and thus influence the success of a prehabilitation intervention [ [20](https://scite.ai/reports/10.4135/9781483348896.n29)]. Our research questions were: How does the gynaecological cancer literature define ‘prehabilitation’?…”

Section: Methodsmentioning

confidence: 99%

“…Improvements in SF-36^ and overall survival [2 years] Strengths: based on the logic change model: the rehabilitation model which has been validated by an expert group Standardised geriatric intervention which is being co-constructed on a multi-professional and multi-disciplinary basis that encompasses the period before surgery, immediately after surgery and discharge Training-Ovary 01 multicenter randomized study comparing neoadjuvant chemotherapy for patients managed for ovarian cancer with or without a connected prehabilitation programme 2021–2024 [ 30 ] France To trial whether a connected prehabilitation programme during NACT will improve physical capacity prior to surgery for advanced ovarian cancer patients 136 patients with advanced ovarian cancer (stage iii-iv) undergoing NACT N = 66 per arm Randomised controlled trial (PROTOCOL) Planned outcomes: primary: to determine whether prehab improves physical conditioning prior to surgery compared with baseline. Outcome measure VO2max Secondary: nutritional status, physical fitness, psychological status Strengths: Follow up period of 5 years Limitations: excludes those without computers and smartphones F4S PREHAB Trial Multimodal intensive Prehabilitation in high impact surgery to reduce postoperative complications 2021–2023 [ [31](https://scite.ai/reports/10.1136/ijgc-2020-002128)] Denmark Understand the effects of prehabilitation on clinical outcomes, the underlying mechanism and cost efficiency of prehabilitation Target N = 2380 Multiple tumour groups including ovarian, endometrial, and vulvar scheduled for high impact surgery Stepped wedge cluster randomised controlled trial (PROTOCOL) Planned outcomes: Primary outcome: Post-operative complications (Clavien-Dindo Score and Comprehensive complication Index) Secondary outcomes: Individual patient level: Length of stay (days), physical fitness (VO 2 max, SQUASH £ questionnaire), nutritional status (body weight, fat free mass PG SGA-SF # ), mental health (SF-36 questionnaire), intervention adherence Mechanistic level: Innate immune response Hospital efficiency level: Costs due to complications, costs due to length of stay, cost-effectiveness Macro-economic level: Changes in patient volumes and shifts in care between 2 nd and 1 st line healthcare Strengths:large multicentre trial Multiple outcome measures Limitations: excluded people with an inability to read or understand Dutch No description of individual components of prehabilitation Impact of a remote Prehabilitation programme in reducing delays to patients having surgery for advanced gynaecological cancer 2021 [ ...…”

Section: Methodsmentioning

confidence: 99%

See 3 more Smart Citations

[Considerations for multimodal prehabilitation in women with gynaecological cancers: a scoping review using realist principles](https://scite.ai/reports/considerations-for-multimodal-prehabilitation-in-zRQZVV4b)

[Rhia Kaur Saggu](https://scite.ai/authors/rhia-kaur-saggu-Wa5L8d)^1^,

[Phillip Barlow](https://scite.ai/authors/phillip-barlow-PlrXaV)^2^,

[John Butler](https://scite.ai/authors/john-butler-1Zbd2X)^3^

et al. 2022

[*BMC Women's Health*](https://scite.ai/journals/1472-6874)
