# Supplementary material for: Prehabilitation for lumbar spinal stenosis: understanding mechanisms and contexts for enhanced engagement—a realist review
Source: Age Ageing. 2025 Oct 24;54(10):afaf311. doi: 10.1093/ageing/afaf311 (PMC12551379; doi:10.1093/ageing/afaf311)
Supplement: Supplementary_materials_afaf311 [file supplementary_materials_afaf311.zip › Supplementary_materials_afaf311_File006.docx]

**Appendix 5: Undermind literature results for CMOC 1**

[Undermind](https://www.undermind.ai/home/)

**Research topic**

*The impact of prehabilitation on patient empowerment and stress reduction in individuals awaiting orthopaedic surgeries.*

**Discuss the results with an expert**

Prehabilitation, particularly when using multimodal approaches that combine education, psychological support, and physical preparation, consistently reduces stress and enhances patient empowerment in individuals awaiting orthopedic surgeries, with key supporting studies including [**1**], [**2**], [**3**], [**7**], and [**9**].

**Report**

**Detailed summary**

Prehabilitation, particularly when using multimodal approaches that combine education, psychological support, and physical preparation, consistently reduces stress and enhances patient empowerment in individuals awaiting orthopedic surgeries, with key supporting studies including [**1**], [**2**], [**3**], [**7**], and [**9**].

**Detailed Summary**:

**Patient Empowerment**: Empowerment-centric prehabilitation methods, such as those implemented in educational programs ([**1**], [**3**]), significantly improve patients' sense of control, confidence, and self-efficacy in managing perioperative tasks. Empowerment-driven education models outperform traditional methods ([**3**]).

**Stress Reduction**: Multimodal programs incorporating mindfulness, relaxation techniques, or psychoeducation effectively reduce preoperative anxiety and stress ([**2**], [**7**], [**9**]), with reductions as high as 45% in stress-related psychometric scores ([**2**]). Psychological support also correlates with long-term improvements in both mental health and surgical readiness ([**7**], [**9**]).

**Multimodal Programs**: Combining physical activity, nutritional counseling, and psychological preparation yields the best outcomes ([**4**], [**19**]). The optimal duration is 4-8 weeks pre-surgery to maximize psychological and physical benefits ([**4**]).

**Orthopedic Focus**: While some studies explicitly address hip and knee arthroplasty ([**1**], [**2**], [**3**], [**7**]), others mix populations, requiring careful application of findings ([**4**], [**19**]).

**Limitations**: Variability in program definitions, lack of standardization, and limited long-term follow-up hinder broader application, though ongoing research is addressing these gaps.

**Categories of papers**

**Categories of Resources Relevant to the Research Topic** **1. Studies Directly Addressing Prehabilitation, Empowerment, and Stress Reduction in Orthopedic Surgeries**

**Description:** These studies explicitly explore the combined impact of prehabilitation on both patient empowerment and stress reduction in patients undergoing orthopedic surgeries.

**References:** [**1**], [**2**], [**3**]

**Details:**

**[1]:** Examines stress reduction through prehabilitation ("Fast Track") encompassing education, active therapy, and empowerment for knee arthroplasty patients.

**[2]:** Investigates stress reduction through empowerment-based education sessions in knee arthroplasty using psychometric and HRV measures with statistically significant results.

**[3]:** Compares empowerment-focused education vs. traditional methods for orthopedic surgeries, finding higher self-efficacy and patient confidence in the empowerment group.

**2. Studies Addressing Stress Reduction via Educational and Psychological Prehabilitation Components**

**Description:** Focuses on psychological or educational interventions in orthopedic patients that aim to reduce preoperative stress (though may not emphasize empowerment equally).

**References:** [**7**], [**5**], [**9**], [**8**]

**Details:**

**[7]:** Psychoeducation reduces anxiety, depression, and pain in hip/knee replacement patients. Significant improvements in stress-related outcomes using HADS scores.

**[5]:** Preoperative education (e.g., “making the unknown familiar”) reduces anxiety in total hip replacement patients by addressing major uncertainties.

**[9]:** Preoperative nurse-led educational and relaxation consultation reduces preoperative anxiety and postoperative pain in hip/knee arthroplasty patients.

**[8]:** Pain management education incorporating relaxation strategies reduced anxiety and improved self-efficacy in musculoskeletal trauma patients awaiting orthopedic surgery.

**3. Studies Addressing Empowerment through Prehabilitation (Orthopedic-Focused)**

**Description:** Emphasizes techniques and interventions designed to improve patient empowerment in orthopedic patient populations. May touch on stress/anxiety indirectly.

**References:** [**6**], [**10**], [**12**]

**Details:**

**[6]:** Pre-admission education in total knee arthroplasty enhances patient confidence, promoting active engagement in health self-management.

**[10]:** Demonstrates greater knowledge and perceived empowerment in hip arthroplasty patients receiving pre-admission education via telephone or written resources.

**[12]:** Links strong self-efficacy to better psychological well-being pre-joint replacement surgery; highlights empowerment via addressing emotional representations.

**4. Multimodal Prehabilitation Interventions with Psychological Elements**

**Description:** Studies on multimodal (physical, psychological, and educational) prehabilitation that indirectly address empowerment or stress, often focusing on orthopedic or related surgeries.

**References:** [**4**], [**19**], [**13**]

**Details:**

**[4]:** Reviews multimodal approaches to prehabilitation in orthopedic surgery and highlights key program structures (e.g., duration, components).

**[19]:** Advocates multi-dimensional prehabilitation for enhanced stress and psychological outcomes while emphasizing integration of exercise, nutrition, and psychological support.

**[13]:** Explores broader prehabilitation concepts, including psychological techniques, with benefits extrapolated to surgical patients.

**5. General Psychological or Educational Prehabilitation (Not Orthopedic-Specific)**

**Description:** Focuses on psycho-educational prehabilitation for other surgical specialties or mixed populations; relevance primarily conceptual in terms of mechanisms or outcomes.

**References:** [**15**], [**16**], [**17**], [**14**], [**18**]

**Details:**

**[15]:** Shows stress reduction using psychological interventions (e.g., mindfulness) within a multimodal prehabilitation program for colorectal surgery.

**[16]:** Reports psychological benefits (anxiety reduction) of preoperative education for spinal surgery; emphasizes individualized approaches.

**[17]:** Scoping review of prehabilitation interventions for spine surgery, including psychological and physical preparation.

**[14]:** Psychoeducation mitigates stress, anxiety, and pain across mixed surgical populations, including knee surgeries. Explores coping mechanisms.

**[18]:** Reviews psychological interventions (e.g., cognitive therapies, relaxation) lowering pre-surgical anxiety and postoperative pain.

**6. Broader Discussions on Prehabilitation Frameworks and Feasibility**

**Description:** Discusses theoretical/methodological frameworks for prehabilitation design and implementation. Relevance is limited to general insights for stress and empowerment strategies.

**References:** [**11**], [**13**], [**19**], [**17**]

**Details:**

**[11]:** Qualitative study highlighting gaps in patient education and engagement in pre-operative pathways for arthroplasty, emphasizing resource barriers.

**[13]:** Review article broadly discussing prehabilitation’s impact on stress and mental health, with indirect relevance to orthopedic surgeries.

**[19]:** Explores the “why, what, how” of multimodal prehabilitation, advocating inclusion of psychological elements for effective patient preparation.

**[17]:** Addresses scalability and logistical challenges in prehabilitation programs, relevant for orthopedic implementation strategies.

**7. Studies Focusing on Specific Outcomes or Measurements Related to Stress and Empowerment**

**Description:** Highlights studies examining specific physiological or psychometric scales to measure prehabilitation outcomes, with relevance to stress or empowerment.

**References:** [**2**], [**7**], [**8**], [**12**]

**Details:**

**[2]:** Uses HRV and psychometric indicators to quantify stress reduction pre/post-empowerment session.

**[7]:** Reports anxiety and pain reductions using HADS and pain scales (VAS) after psychoeducation in knee/hip replacement patients.

**[8]:** Employs quantitative tools (e.g., coping strategy measures) to evaluate self-efficacy and stress relief from prehabilitation education.

**[12]:** Demonstrates positive associations between self-efficacy and psychological well-being using standardized self-efficacy and emotional representation scales.

**Final Notes:**

References in **Categories 1, 2, and 3** are the most focused on your topic, covering empowerment and stress reduction in orthopedic surgery prehabilitation.

**Category 4** provides essential context for multimodal program design but with less direct attention to psychological benefits or orthopedic focus.

Other references contribute indirectly to understanding mechanisms (e.g., stress-pain interaction, feasibility) or broader dimensions of prehabilitation relevance.

**Timeline and citation network**

**Timeline and Historical Development of Concepts:**

**1998-2010**: Foundational focus on **patient empowerment and education**:

**[3] (1998)**: Introduced empowerment-based education for orthopedic surgery, emphasizing improved self-efficacy and confidence in perioperative tasks. This set the stage for later studies linking psychological empowerment to surgical outcomes.

**[5] (2003)**: Highlighted anxiety reduction through educational programs by fostering familiarity with surgical settings, further advocating psychoeducation as a critical preoperative intervention.

**[6, 8] (2010)**: Strengthened the connection between tailored education programs and enhanced outcomes (e.g., anxiety reduction, self-efficacy). Reiterated the need for individualized patient strategies.

**2011-2014**: Emergence of stress and empowerment as integrated goals:

**Fast Track Protocol in Barcelona** ([**1**], [**2**]): Established education-driven empowerment to reduce stress preoperatively, incorporating physiological measures like heart rate variability. This marked a shift toward objective measurement of psychological outcomes.

**[10] (2010)**: Expanded analysis to include patient engagement models, extending empowerment discussions into pre-admission education.

**2017-2019**: Multimodal prehabilitation gains prominence:

**[4], [19] (2019)**: Systematic reviews highlighted multimodal prehabilitation’s superiority, combining physical, psychological, and educational elements for orthopedic patients. Timing and program duration (4-8 weeks) were recognized as critical factors.

**[7], [9] (2019)**: Explored the role of preoperative psychoeducation in reducing stress and anxiety, focusing on resource-intensive interventions (e.g., therapeutic education paired with relaxation).

**2020-Present**: Broadening multimodal and population-specific evidence:

**[9], [19]**: Continued exploring structured multimodal programs but identified gaps in standardized protocols and feasibility in scaling such interventions.

**[13] (2022)**: Shifted focus to broader surgical populations, emphasizing the physiological and psychological benefits of prehabilitation but with weaker orthopedic-specific evidence.

**Clusters of Research Groups and Impactful Contributions:**

**Fast Track Barcelona Team ([1], [2])**:

Pioneers integrating psychological empowerment into prehabilitation protocols for knee arthroplasty patients, providing clear links between stress markers and education-driven empowerment.

**Empowerment Education Advocates ([3], [5], [6])**:

Established the theoretical foundation for empowerment-based teaching approaches in orthopedic settings, influencing future psychological interventions.

**Multimodal Optimization Leaders ([4], [19])**:

Advocated multimodal program frameworks incorporating physical, psychological, and educational components, emphasizing tailored protocols for optimal adherence and outcomes.

**Recent Advancements ([7], [9])**:

Current work focuses on integrating advanced psychoeducational techniques (e.g., therapeutic relaxation) to address anxiety and pain in hip/knee arthroplasty, albeit with challenges in resource allocation.

**Key Takeaways:**

Progression has evolved from **unimodal education-focused approaches** (pre-2010) to **empowerment-based models incorporating stress reduction** (2011-2019), and now emphasizes **comprehensive multimodal prehabilitation** with tailored protocols (post-2020).

Research clusters have advanced understanding by combining educational, psychological, and physiological methodologies, though long-term data and standardization remain areas for development.

**Discovery progress: ~51.7% complete (~4-12 papers found)**

So far, I've closely analyzed 140 of the most promising papers, and I've found **~4-12** papers with relevant abstracts, which is probably **~51.7%** of all that exist.

85.9%

2017

[**[1] Do educational and empowerment sessions reduce stress levels before knee arthroplasty?**](https://www.semanticscholar.org/paper/88406d23be2caed2e5bb3e1aa40d6a8475a1ac1b)M. Nuevo, ..., and T. Faura[Journal Not Provided](https://www.semanticscholar.org/paper/88406d23be2caed2e5bb3e1aa40d6a8475a1ac1b)2017 - 2 citations - [Show abstract](https://www.undermind.ai/query_app/display_one_search/c7b3536bef44f0aedbd382cd5fa2d4d877f15a8042329504653aff77928343ab/) - [Cite](javascript:void(0);)

**Provides insights into patient education and empowerment reducing stress before knee arthroplasty surgery.** Focuses on a "Rapid Recovery" protocol integrating education, team coordination, and active physical therapy for knee surgery patients. Relevant for stress reduction and empowerment in orthopaedic surgery, but lacks mention of comprehensive multimodal prehabilitation programs.

80.7%

0.1

2014

[**[2] Patient empowerment and stress reduction**](https://doi.org/10.1109/IBERSENSOR.2014.6995560)Adriana Arza Valdés, ..., and J. Aguiló[2014 IEEE 9th IberoAmerican Congress on Sensors](https://doi.org/10.1109/IBERSENSOR.2014.6995560)2014 - 1 citations - [Show abstract](https://www.undermind.ai/query_app/display_one_search/c7b3536bef44f0aedbd382cd5fa2d4d877f15a8042329504653aff77928343ab/) - [Cite](javascript:void(0);)

**Provides insights on patient empowerment and stress reduction before knee arthroplasty.**Examines effects of an educational session on stress using psychometric tests and HRV, showing significant stress reduction in orthopedic patients.Focuses on single-modality (educational) intervention, not comprehensive prehabilitation, potentially limiting direct applicability to multimodal prehabilitation programs.

80.4%

1998

[**[3] Increasing Self‐Efficacy Through Empowerment: Preoperative Education for Orthopaedic Patients**](https://doi.org/10.1097/00006416-199807000-00009)T. Pellino, ..., and Judith Broad[Orthopaedic Nursing](https://doi.org/10.1097/00006416-199807000-00009)1998 - 119 citations - [Show abstract](https://www.undermind.ai/query_app/display_one_search/c7b3536bef44f0aedbd382cd5fa2d4d877f15a8042329504653aff77928343ab/) - [Cite](javascript:void(0);)

**Provides evidence on empowerment-focused preoperative education improving self-efficacy in orthopaedic surgery patients.** Compared empowerment-based vs. traditional teaching; found higher self-efficacy and confidence in perioperative tasks with empowerment methods. Focuses on education as a standalone intervention, not multimodal prehabilitation, and does not address stress reduction explicitly.

80.3%

2019

[**[4] Effectiveness of prehabilitation for patients undergoing orthopaedic surgery: protocol for a systematic review and meta-analysis**](https://doi.org/10.1136/bmjopen-2019-031119)A. Punnoose, ..., and A. Rushton[BMJ Open](https://doi.org/10.1136/bmjopen-2019-031119)2019 - 3 citations - [Show abstract](https://www.undermind.ai/query_app/display_one_search/c7b3536bef44f0aedbd382cd5fa2d4d877f15a8042329504653aff77928343ab/) - [Cite](javascript:void(0);) - [PDF](https://bmjopen.bmj.com/content/bmjopen/9/11/e031119.full.pdf)

**Evaluates prehabilitation's effectiveness in orthopaedic surgery patients.**Focuses on physical fitness, function, program components, duration, and frequency, but doesn't explicitly mention empowerment or stress reduction.Systematic review protocol; relevance depends on whether psychological outcomes are included in later analyses (not clear in abstract).

73.2%

2003

[**[5] Reducing anxiety by pre-operative education: make the future familiar.**](https://doi.org/10.1002/OTI.191)Nikki Spalding[Occupational therapy international](https://doi.org/10.1002/OTI.191)2003 - 120 citations - [Show abstract](https://www.undermind.ai/query_app/display_one_search/c7b3536bef44f0aedbd382cd5fa2d4d877f15a8042329504653aff77928343ab/) - [Cite](javascript:void(0);)

**Shows that pre-operative education reduces anxiety in hip replacement patients.**Explores how education familiarizes patients with future experiences, staff, and environments, thereby decreasing pre-surgical anxiety.Focuses solely on stress reduction, not empowerment or multimodal prehabilitation, and is specific to hip replacement surgeries.

67.5%

2010

[**[6] Patient Education and Involvement in Care.**](https://www.semanticscholar.org/paper/a5588b4a6a5625350461280280722222db6596a2)Linda R. Andiric[Journal Not Provided](https://www.semanticscholar.org/paper/a5588b4a6a5625350461280280722222db6596a2)2010 - 1 citations - [Show abstract](https://www.undermind.ai/query_app/display_one_search/c7b3536bef44f0aedbd382cd5fa2d4d877f15a8042329504653aff77928343ab/) - [Cite](javascript:void(0);)

**Shows the impact of preadmission education on patient empowerment in orthopedic surgery (knee arthroplasty).**Highlights improved outcomes when education fosters confidence, control, and active involvement in pre- and post-surgery care.Lacks comprehensive prehabilitation programs or explicit focus on stress reduction; primarily addresses empowerment through education for knee surgery.

59.8%

2019

[**[7] The Effect of Psycho-Education on Depression, Anxiety and Pain for Patients Undergoing Hip And Knee Replacement**](https://doi.org/10.21608/asnj.2019.58110)Hanan Maghawry[Assiut Scientific Nursing Journal](https://doi.org/10.21608/asnj.2019.58110)2019 - 0 citations - [Show abstract](https://www.undermind.ai/query_app/display_one_search/c7b3536bef44f0aedbd382cd5fa2d4d877f15a8042329504653aff77928343ab/) - [Cite](javascript:void(0);)

**Provides evidence on psycho-education reducing anxiety in orthopedic surgery patients.**Investigates the impact of preoperative psycho-education on anxiety, depression, and pain in hip and knee replacement patients.Focuses on a single-modality intervention (psycho-education), not multimodal prehabilitation, and does not address patient empowerment explicitly.

55.6%

2010

[**[8] Effectiveness of an educational intervention on levels of pain, anxiety and self-efficacy for patients with musculoskeletal trauma.**](https://doi.org/10.1111/j.1365-2648.2010.05273.x)E. Wong, ..., and S. Chair[Journal of advanced nursing](https://doi.org/10.1111/j.1365-2648.2010.05273.x)2010 - 101 citations - [Show abstract](https://www.undermind.ai/query_app/display_one_search/c7b3536bef44f0aedbd382cd5fa2d4d877f15a8042329504653aff77928343ab/) - [Cite](javascript:void(0);)

**Provides evidence on preoperative education improving anxiety and self-efficacy in musculoskeletal trauma patients awaiting orthopaedic surgery.**Demonstrates lower anxiety and higher self-efficacy from a 30-minute educational intervention emphasizing pain, coping, and relaxation techniques.Focuses solely on education, not comprehensive prehabilitation; does not directly address patient empowerment or long-term stress reduction.

55.4%

2023

[**[9] Effets préliminaires d’une consultation préopératoire infirmière auprès des patients devant subir une arthroplastie de la hanche ou du genou : une étude préexpérimentale**](https://doi.org/10.3917/rsi.151.0099)Marie-Paule Bell, ..., and Justine Zehr[Recherche en soins infirmiers](https://doi.org/10.3917/rsi.151.0099)2023 - 0 citations - [Show abstract](https://www.undermind.ai/query_app/display_one_search/c7b3536bef44f0aedbd382cd5fa2d4d877f15a8042329504653aff77928343ab/) - [Cite](javascript:void(0);)

**Provides insights on preoperative interventions reducing anxiety in hip/knee replacement patients.**Evaluates a preoperative nursing consultation using education and relaxation, showing significant anxiety reductions pre-surgery and links to postoperative outcomes.Focuses on anxiety reduction but lacks emphasis on broader patient empowerment or comprehensive multimodal prehabilitation programs.

54.1%

2010

[**[10] Pre-admission education in surgical rheumatology nursing: towards greater patient empowerment.**](https://doi.org/10.1111/j.1365-2702.2010.03347.x)K. Johansson, ..., and S. Salanterä[Journal of clinical nursing](https://doi.org/10.1111/j.1365-2702.2010.03347.x)2010 - 20 citations - [Show abstract](https://www.undermind.ai/query_app/display_one_search/c7b3536bef44f0aedbd382cd5fa2d4d877f15a8042329504653aff77928343ab/) - [Cite](javascript:void(0);)

**Compares education methods aimed at empowering rheumatoid arthritis patients pre-hip arthroplasty.**Assesses impact on knowledge, certainty, and learning experience but focuses on standalone education, not comprehensive prehabilitation.Limited relevance: addresses empowerment but lacks stress reduction or multimodal prehabilitation for orthopedic patients.

42.9%

2020

[**[11] 59 A Qualitative Study Evaluating Patient Education in the Pre-Operative Pathway for Elective Hip and Knee Arthroplasty at A Major London Teaching Hospital**](https://doi.org/10.1093/ageing/afz186.13)Rami Abbass, ..., and E. Tonner[Age and Ageing](https://doi.org/10.1093/ageing/afz186.13)2020 - 0 citations - [Show abstract](https://www.undermind.ai/query_app/display_one_search/c7b3536bef44f0aedbd382cd5fa2d4d877f15a8042329504653aff77928343ab/) - [Cite](javascript:void(0);)

**Explores patient education in preoperative pathways for hip/knee arthroplasty.**Highlights issues with ineffective patient engagement causing distress, but focuses on standalone education rather than multimodal prehabilitation.Does not discuss psychological outcomes like stress reduction or empowerment, limiting relevance to the topic's focus on prehabilitation.

38.6%

2016

[**[12] The associations of illness perceptions and self-efficacy with psychological well-being of patients in preparation for joint replacement surgery**](https://doi.org/10.1080/13548506.2015.1115109)E. Magklara and V. Morrison[Psychology, Health & Medicine](https://doi.org/10.1080/13548506.2015.1115109)2016 - 9 citations - [Show abstract](https://www.undermind.ai/query_app/display_one_search/c7b3536bef44f0aedbd382cd5fa2d4d877f15a8042329504653aff77928343ab/) - [Cite](javascript:void(0);)

**Explores psychological well-being factors in patients awaiting joint replacement surgery.**Examines how illness perceptions and self-efficacy relate to pre-surgical emotional well-being, highlighting modifiable psychological factors.Does not specifically address prehabilitation or stress reduction interventions, but focuses on psychological constructs relevant to patient empowerment.

38.0%

2022

[**[13] Prehabilitation in Patients before Major Surgery: A Review Article**](https://doi.org/10.31729/jnma.7545)Pawan Shakya and Sagar Poudel[JNMA: Journal of the Nepal Medical Association](https://doi.org/10.31729/jnma.7545)2022 - 13 citations - [Show abstract](https://www.undermind.ai/query_app/display_one_search/c7b3536bef44f0aedbd382cd5fa2d4d877f15a8042329504653aff77928343ab/) - [Cite](javascript:void(0);)

**Provides a general overview of prehabilitation and its benefits.**Discusses physical and nutritional interventions, stress response reduction, and mental health improvements, but lacks focus on patient empowerment or orthopaedic contexts.Does not explicitly address psychological outcomes like stress reduction in orthopaedic patients or multimodal prehabilitation specifics.

35.0%

2018

[**[14] COPING, HOSPITAL ANXIETY AND DEPRESSION AND PAIN EXPERIENCE IN ELECTIVE SURGERY PATIENTS: ROLE OF PSYCHO-EDUCATIONAL INTERVENTIONS**](https://www.semanticscholar.org/paper/2b68e564419d18fe73ea775121de881df3c46c49)Usha Chivukula and Durgesh Nandinee[Paripex Indian Journal Of Research](https://www.semanticscholar.org/paper/2b68e564419d18fe73ea775121de881df3c46c49)2018 - 1 citations - [Show abstract](https://www.undermind.ai/query_app/display_one_search/c7b3536bef44f0aedbd382cd5fa2d4d877f15a8042329504653aff77928343ab/) - [Cite](javascript:void(0);)

**Provides general insights on psycho-educational interventions reducing anxiety and depression in elective surgery patients.**Focuses on improving coping, mitigating anxiety/depression, and reducing pain, but includes various surgeries beyond orthopaedic contexts.Does not examine comprehensive prehabilitation or patient empowerment; only partially overlaps with stress reduction focus in orthopaedics.

21.1%

2020

[**[15] The effect of trimodal prehabilitation on the physical and psychological health of patients undergoing colorectal surgery: a randomised clinical trial**](https://doi.org/10.1111/anae.15215)A. Fulop, ..., and B. Banky[Anaesthesia](https://doi.org/10.1111/anae.15215)2020 - 59 citations - [Show abstract](https://www.undermind.ai/query_app/display_one_search/c7b3536bef44f0aedbd382cd5fa2d4d877f15a8042329504653aff77928343ab/) - [Cite](javascript:void(0);)

**Shows the impact of trimodal prehabilitation on physical and psychological health in colorectal surgery.**Examines a 4-week program including physical, nutritional, and psychological interventions, measuring anxiety, depression, and functional outcomes.Focuses on colorectal, not orthopedic, surgeries; psychological outcomes are measured but patient empowerment isn't explicitly addressed.

20.6%

2019

[**[16] The Effect of Preoperative Education on Psychological, Clinical and Economic Outcomes in Elective Spinal Surgery: A Systematic Review**](https://doi.org/10.3390/healthcare7010048)Louise C. Burgess, ..., and T. Wainwright[Healthcare](https://doi.org/10.3390/healthcare7010048)2019 - 60 citations - [Show abstract](https://www.undermind.ai/query_app/display_one_search/c7b3536bef44f0aedbd382cd5fa2d4d877f15a8042329504653aff77928343ab/) - [Cite](javascript:void(0);) - [PDF](https://www.mdpi.com/2227-9032/7/1/48/pdf?version=1553159812)

**Provides evidence on preoperative education's psychological impact for spinal surgery patients.**Shows limited evidence that preoperative education reduces anxiety, depression, and fear-avoidance while improving perceived preparation and knowledge.Focuses on education alone, not comprehensive prehabilitation, and is specific to spinal surgery, not general orthopaedic procedures.

19.0%

2023

[**[17] Prehabilitation for spine surgery: A scoping review**](https://doi.org/10.1002/pmrj.12956)James E. Eubanks, ..., and Michael J. Schneider[PM&R](https://doi.org/10.1002/pmrj.12956)2023 - 6 citations - [Show abstract](https://www.undermind.ai/query_app/display_one_search/c7b3536bef44f0aedbd382cd5fa2d4d877f15a8042329504653aff77928343ab/) - [Cite](javascript:void(0);) - [PDF](https://cris.maastrichtuniversity.nl/files/187608020/Smeets-2023-Prehabilitation-for-spine-surgery-A.pdf)

**Provides a review of prehabilitation for elective spine surgeries.**Explores physical and psychological outcomes, including general education components, though focus is on spine surgery, not orthopedics broadly.Relevance is limited: does not explicitly address empowerment, stress reduction, or non-spine orthopedic preoperative contexts.

18.6%

2020

[**[18] Primary Prevention of Chronic Pain**](https://www.semanticscholar.org/paper/0b19c57734ed4511b34cd1c6488e06f89da97efe)No author found[Journal Not Provided](https://www.semanticscholar.org/paper/0b19c57734ed4511b34cd1c6488e06f89da97efe)2020 - 0 citations - [Show abstract](https://www.undermind.ai/query_app/display_one_search/c7b3536bef44f0aedbd382cd5fa2d4d877f15a8042329504653aff77928343ab/) - [Cite](javascript:void(0);)

**Shows limited evidence on prehabilitation’s effects on psychological outcomes like stress and anxiety.**Mentions that prehabilitation with psychological components (e.g., relaxation, cognitive strategies) may slightly reduce negative affect and anxiety post-surgery.Does not focus specifically on orthopaedic surgeries or patient empowerment; evidence quality is described as low to very low.

17.6%

2019

[**[19] Multi‐modal prehabilitation: addressing the why, when, what, how, who and where next?**](https://doi.org/10.1111/anae.14505)C. Scheede-Bergdahl, ..., and F. Carli[Anaesthesia](https://doi.org/10.1111/anae.14505)2019 - 142 citations - [Show abstract](https://www.undermind.ai/query_app/display_one_search/c7b3536bef44f0aedbd382cd5fa2d4d877f15a8042329504653aff77928343ab/) - [Cite](javascript:void(0);) - [PDF](https://onlinelibrary.wiley.com/doi/pdfdirect/10.1111/anae.14505)

**Provides a review of multimodal prehabilitation's importance for pre-surgical preparation.**Highlights how combining physical, nutritional, and psychological components enhances overall prehabilitation effectiveness, particularly adherence and response to interventions.Does not specify orthopaedic surgeries, patient empowerment, or stress reduction, limiting direct relevance to the desired topic.

13.4%

2019

[**[20] Preoperative Optimization of Chronic Pain Patients Undergoing Surgery**](https://doi.org/10.1097/BTO.0000000000000433)P. Gulur and Amanda H Nelli[Techniques in Orthopaedics](https://doi.org/10.1097/BTO.0000000000000433)2019 - 1 citations - [Show abstract](https://www.undermind.ai/query_app/display_one_search/c7b3536bef44f0aedbd382cd5fa2d4d877f15a8042329504653aff77928343ab/) - [Cite](javascript:void(0);)

12.5%

0.0

2018

[**[21] Understanding Prehabilitation**](https://www.semanticscholar.org/paper/753229b7a39cb5e2bf589ee57d9e276641b79177)D. Milliken and Dr Nick Schofield[Journal Not Provided](https://www.semanticscholar.org/paper/753229b7a39cb5e2bf589ee57d9e276641b79177)2018 - 0 citations - [Show abstract](https://www.undermind.ai/query_app/display_one_search/c7b3536bef44f0aedbd382cd5fa2d4d877f15a8042329504653aff77928343ab/) - [Cite](javascript:void(0);)

11.8%

2004

[**[22] Pre-operative education for hip or knee replacement.**](https://doi.org/10.1002/14651858.CD003526.PUB2)Steven McDonald, ..., and Sally Green[The Cochrane database of systematic reviews](https://doi.org/10.1002/14651858.CD003526.PUB2)2004 - 216 citations - [Show abstract](https://www.undermind.ai/query_app/display_one_search/c7b3536bef44f0aedbd382cd5fa2d4d877f15a8042329504653aff77928343ab/) - [Cite](javascript:void(0);) - [PDF](https://research.monash.edu/files/272278172/272276093_oa.pdf)

10.8%

2020

[**[23] Surgical Prehabilitation: Systematic Review**](https://doi.org/10.20431/2455-572x.0602002)A. A. Rodríguez, ..., and A. M. Benítez[ARC Journal of Surgery](https://doi.org/10.20431/2455-572x.0602002)2020 - 0 citations - [Show abstract](https://www.undermind.ai/query_app/display_one_search/c7b3536bef44f0aedbd382cd5fa2d4d877f15a8042329504653aff77928343ab/) - [Cite](javascript:void(0);)
