# Supplementary material for: Prehabilitation for lumbar spinal stenosis: understanding mechanisms and contexts for enhanced engagement—a realist review
Source: Age Ageing. 2025 Oct 24;54(10):afaf311. doi: 10.1093/ageing/afaf311 (PMC12551379; doi:10.1093/ageing/afaf311)
Supplement: Supplementary_materials_afaf311 [file supplementary_materials_afaf311.zip › Supplementary_materials_afaf311_File007.docx]

**Appendix 6: Relevance Screening tool for Literature search One (Ti/Ab screen)**

| Relevance | Criteria |
| --- | --- |
| 5- Highly relevant | - **Condition:** Related to lumbar spinal stenosis and/or neurogenic claudication - **Surgical Pathway:** Focuses on prehabilitation within a surgical pathway for neurogenic claudication and/or lumbar spinal stenosis in adults over 50 years old. - **Prehabilitation Framework**: Addresses all three elements of the trimodal framework of prehabilitation (exercise, nutrition, and psychological intervention). - **Evaluation:** Evaluates the effectiveness of prehabilitation intervention, explores the experience of implementing/participating in prehabilitation or describes the process of developing a prehabilitation intervention for this patient group. |
| 4 – Probably relevant | - **Condition:** Related to lumbar spinal stenosis and/or neurogenic claudication - **Surgical Pathway:** Focuses on prehabilitation within a surgical pathway for neurogenic claudication and/or lumbar spinal stenosis in adults over 50 years old. - **Prehabilitation Framework**: Addresses one or two elements of the trimodal framework of prehabilitation but not all three (exercise, nutrition, and psychological intervention). - **Evaluation:** Evaluates the effectiveness of prehabilitation intervention, explores the experience of implementing/participating in prehabilitation or describes the process of developing a prehabilitation intervention for this patient group |
| 3 – Possibly relevant | - **Condition:** Related to degenerative lumbar spine conditions - **Surgical Pathway:** Focuses on prehabilitation within a surgical pathway for degenerative lumbar spinal disease in adults over 18 years old. - **Prehabilitation Framework**: Addresses one or two elements of the trimodal framework of prehabilitation but not all three (exercise, nutrition, and psychological intervention). OR it is unclear as to the components of the prehabilitation programme OR it refers to ERAS programme - **Evaluation:** Evaluates the effectiveness of prehabilitation intervention, explores the experience of implementing/participating in prehabilitation or describes the process of developing a prehabilitation intervention for this patient group |
| 2 – Less relevant | - **Condition:** Not related to the lumbar spine OR not related to degenerative lumbar spine conditions - **Surgical Pathway:** Focuses on prehabilitation within a surgical pathway for any condition. - **Prehabilitation Framework**: Addresses one or two elements of the trimodal framework of prehabilitation but not all three (exercise, nutrition, and psychological intervention). OR it is unclear as to the components of the prehabilitation programme - **Evaluation:** Reports on the effectiveness of the prehabilitation intervention or evaluates the experience of implementing prehabilitation in this setting. |
| 1 – Likely irrelevant | - **Condition:** Not related to the lumbar spine OR not related to degenerative lumbar spine conditions OR related to adolescents (<18 y.o) - **Surgical Pathway:** Focuses on prehabilitation outside of a surgical context. - **Prehabilitation Framework**: primary focus is on perioperative care, with prehabilitation mentioned only as a component within a broader perioperative programme OR does not relate to prehabilitation. - **Evaluation:** The evaluation or report focuses on general preoperative care, not specifically on the design, implementation, or outcomes of a dedicated prehabilitation intervention. |
