# Supplementary material for: Prehabilitation for lumbar spinal stenosis: understanding mechanisms and contexts for enhanced engagement—a realist review
Source: Age Ageing. 2025 Oct 24;54(10):afaf311. doi: 10.1093/ageing/afaf311 (PMC12551379; doi:10.1093/ageing/afaf311)
Supplement: Supplementary_materials_afaf311 [file supplementary_materials_afaf311.zip › Supplementary_materials_afaf311_File008.docx]

**Appendix 7: Relevance, Richness and Rigour screening tool for Literature Search One**

(Full text screen)

**Revised screen**

|  | **Relevance** | **Richness** | **Rigour** |
| --- | --- | --- | --- |
| **High** | Directly addresses the topic and research question. Makes significant contributions to theory development by proposing new models, frameworks, or insights | Offers a thorough and detailed analysis. Provides a variety of perspectives and evidence | Highly credible source with strong methodology. Methods are transparent, appropriate, and well-executed. |
| **Moderate** | Somewhat relevant to the topic. May offer contributions to theory development but they are limited or lack depth. | Provides some detail but lacks depth or comprehensiveness. May have limited data or examples. | Credible source with generally sound methodology. May have some limitations in methodology. |
| **Low** | Limited relevance to the topic. Offers little to no contribution to theory development. Information is tangential or not helpful. | Offers minimal detail or lacks sufficient information | Source has questionable credibility or significant methodological flaws. |
