# Supplementary material for: Prehabilitation for lumbar spinal stenosis: understanding mechanisms and contexts for enhanced engagement—a realist review
Source: Age Ageing. 2025 Oct 24;54(10):afaf311. doi: 10.1093/ageing/afaf311 (PMC12551379; doi:10.1093/ageing/afaf311)
Supplement: Supplementary_materials_afaf311 [file supplementary_materials_afaf311.zip › Supplementary_materials_afaf311_File009.docx]

**Appendix 8: Relevance, Richness and Rigour screening tool for Literature Search Two**

(Full text screen)

| **Relevance** | **Criteria** |
| --- | --- |
| High | - Provides strong empirical evidence directly relevant to the CMOC. - Offers deep insights into the underlying mechanisms or contexts of the CMOC. - Contributes significantly to the understanding, refinement, or expansion of the CMOC. - May be relevant to multiple CMOCs |
| Moderate | - Provides sufficient empirical evidence relevant to the CMOC. - Offers some insights into the underlying mechanisms or contexts of the CMOC. - May be relevant to multiple CMOCs, but the depth of analysis may be limited. - May lack explicit reference to the underlying theory. |
| Low | - Provides limited or weak empirical evidence related to the CMOC. - Offers minimal insights into the underlying mechanisms or contexts. - Has a limited impact on the understanding or refinement of the CMOC. - May be tangential to the CMOC or only indirectly related. |
| **Richness** | **Criteria** |
| High | - Provides detailed and nuanced empirical data that directly supports or refutes the CMOC - Offers deep insights into the underlying mechanisms or contexts of the CMOC - Is conceptually rich, with well-grounded and clearly described theories and concepts. |
| Moderate | - Provides sufficient empirical data relevant to the CMOC - Offers some insights into the underlying mechanisms or contexts of the CMOC - Is conceptually thick, with a rich description of the program or intervention but without explicit reference to the underlying theory. |
| Low | - Provides limited or superficial empirical data related to the CMOC - Offers minimal insights into the underlying mechanisms or context - Is conceptually thin, with weak program descriptions and difficulty discerning the underlying theory. |
| **Rigour** | **Criteria** |
| High | - **Data Source Credibility:** The data source is highly credible and trustworthy. Any potential biases or limitations are clearly acknowledged and addressed. - **Methodological Appropriateness:** The methods used to generate the data are highly appropriate for the research question and context. The data collection and analysis methods are clearly described and justified. Any limitations in the methods are acknowledged and their potential impact on the findings is considered. - **Overall Judgment:** The reviewer has high confidence in the trustworthiness and validity of the data and its relevance to the CMOC being explored. The CCAT may have been used to inform this judgement, but the ultimate decision rests on the expert judgement of the reviewer. |
| Moderate | - **Data Source Credibility:** The data source is generally credible and trustworthy, but there may be some minor concerns about potential biases or limitations. These concerns are acknowledged and considered in the interpretation of the data. - **Methodological Appropriateness:** The methods used to generate the data are generally appropriate, but there may be some minor limitations or inconsistencies. - **Overall Judgment:** The reviewer has reasonable confidence in the trustworthiness and validity of the data, though some uncertainties remain. The data provides reasonable support for the conclusions drawn, but further supporting evidence might be beneficial. The CCAT may have been used to inform this judgement, but the ultimate decision rests on the expert judgement of the reviewer. |
| Low | - **Data Source Credibility:** The data source has significant concerns regarding credibility or trustworthiness. There may be evidence of bias, lack of expertise, or questionable data collection/analysis practices. - **Methodological Appropriateness:** The methods used to generate the data are significantly flawed or inappropriate for the research question and context. There may be a lack of clear description or justification of the methods, or evidence of significant methodological weaknesses. - **Overall Judgment:** The reviewer has low confidence in the trustworthiness and validity of the data. The data provides weak or insufficient support for the conclusions drawn. The CCAT may have been used to inform this judgement, but the ultimate decision rests on the expert judgement of the reviewer. The data may be deemed too flawed to contribute meaningfully to the review. |
