# Supplementary material for: Prehabilitation for lumbar spinal stenosis: understanding mechanisms and contexts for enhanced engagement—a realist review
Source: Age Ageing. 2025 Oct 24;54(10):afaf311. doi: 10.1093/ageing/afaf311 (PMC12551379; doi:10.1093/ageing/afaf311)
Supplement: Supplementary_materials_afaf311 [file supplementary_materials_afaf311.zip › Supplementary_materials_afaf311_File010.docx]

**Appendix 9: Data Extraction Form (example)**

Data extraction form Reference: Francis-Coad 2021

Literature search 2

**PRESS**

Development of a prehabilitation intervention for people with lumbar spinal stenosis undergoing surgery

| **Title:**  Partnering with patients to design a prehabilitation program for optimizing the patient experience through general surgery |
| --- |

| **Year:**  2021 | **Country:**  Australia | **Relevance:**  High | **Richness:**  High | **Rigour**  High |
| --- | --- | --- | --- | --- |

| CMOCs | 3, 6, 12, 13 |
| --- | --- |

| **Principal findings:**  Patients confirmed the pre-surgical period as an opportunity to engage in preparing physically and psychologically for surgery and recovery. Patients’ experiences of the surgical journey identified gaps that impacted their capability, opportunity, and motivation to effectively prepare and rehabilitate that could be addressed by a multimodal prehabilitation program. Intervention options at patient and policy level were identified for trial to enhance the patient experience of general surgery. |
| --- |

| **Notes:**  **CMOC 6**  p.137 *“Patients interacted with many health professional staff prior to their surgery, specifically anaesthetists, surgeons, physicians, clinic nurses, pharmacists and physiotherapists. The type of information patients received pre-operatively varied, even allowing for different surgical procedures. At primary clinic appointments, all patients felt they were well informed regarding understanding their surgical procedure, anaesthesia and pain management that instilled feelings of empowerment. This enabled them to be prepared to work with their treating team to optimise their recovery. P7“I saw a lot of people and got a lot of answers…the surgeon was brilliant he drew diagrams to explain (the procedure)…I knew what I would wake up with in relation to monitoring, tubes in and out of my body, pain medication…I found that very helpful.”*  This finding *strongly supports* and *refines* CMOC 6. The theory argues that consistent messaging from healthcare professionals (C) prevents confusion, fear, and mistrust (M), thus protecting the program's value (O). The finding about patients interacting with *many* healthcare professionals (anaesthetists, surgeons, physicians, nurses, pharmacists, physiotherapists) and receiving *varied* information directly speaks to the challenge of maintaining consistency in such a complex care pathway. The fact that information varied "even allowing for different surgical procedures" suggests that while some variation might be expected, the level of variation described points to potential inconsistencies. The finding underscores the *need* for consistent messaging, as highlighted by CMOC 6.  The finding *refines* CMOC 6 by highlighting the *multiple sources* of information and the potential for *varied messages* across different healthcare professionals. It's not just about *one* professional giving inconsistent information; it's about the *cumulative effect* of multiple professionals potentially contributing to confusion if their messages aren't aligned. The quote about the surgeon using diagrams to explain the procedure shows *how* clear communication can be effective, but it also implies that *not all* professionals may communicate with the same level of clarity or detail, which could contribute to inconsistencies. This highlights the need for *coordinated communication* across the entire healthcare team.  **CMOC 12**  p.139 *“A key opportunity patients felt was missing was the opportunity to share advice from other patients who had been through similar surgical experiences to assist their recovery.”*  p.139 *“Patients strongly concurred that their family highly motivated them to optimise their recovery. Families were described as providing the essential support that enabled patients to successfully complete the journey from pre-op to final recovery,”*  This finding *strongly supports* and *refines* CMOC 12. The theory focuses on online peer groups (C) as a source of support and reassurance (O) through shared experiences and guidance (M). The finding about patients wanting to share advice with others who have had similar surgeries directly supports the core idea of peer support being valuable. The quotes from patients P7 and P12, expressing a desire for "first-hand experience" and "watching a video of a patient describing their surgical experience," clearly illustrate the need for and perceived value of peer interaction. The "frequent head nodding and statements of 'yes, agreed'" from other patients further reinforces the widespread agreement on this point.  The finding *refines* CMOC 12 in several key ways:   - **It emphasizes the importance of *lived experience* and *practical advice*:** The patients' desire for "first-hand experience" and "what I think I should have done" highlights the unique value of advice from someone who has "been there." It suggests that practical tips and insights from peers are highly sought after. - **It suggests that *various formats* for sharing peer experiences are valuable, not just online groups:** While CMOC 12 focuses on *online* groups, the finding mentions videos as a potentially valuable format. This implies that a range of methods for sharing peer experiences (videos, written testimonials, in-person meetings, online forums) could be beneficial. - **It highlights the role of *family support* alongside peer support:** While CMOC 12 focuses on peer groups, the finding emphasizes the crucial role of family in motivating and supporting patients throughout their recovery. This suggests that family support is another important element to consider in prehabilitation programs.   Therefore, this finding strongly supports CMOC 12 by demonstrating the value of peer support. It also *refines* the theory by highlighting the importance of lived experience and practical advice, suggesting that various formats for sharing peer experiences are valuable, and emphasizing the role of family support alongside peer support. The refined theory might suggest that prehabilitation programs should facilitate access to peer support through a variety of methods (online forums, videos, in-person groups, etc.) and also recognize and support the role of family in patient recovery.  **CMOC 13**  p.140 *“Two patients felt there was a degree of oversight in being on a waiting list for several months; the first P8 stated, “I went on the waiting list…just hanging around…it seemed to take for ever…you think nothing’s ever going to get done,” the second lamented P1 “you feel forgotten, like you don’t matter and that what’s wrong with you isn’t important.”*  This finding *strongly supports* and *refines* CMOC 13. The theory states that regular telephone follow-ups (C) mitigate the sense of abandonment (O) by providing a platform for questions and support. The finding, with patients feeling "forgotten," like "nothing's ever going to get done," and that "what's wrong with you isn't important," directly illustrates the sense of abandonment the theory describes. These quotes are powerful examples of the negative emotional impact of long wait times.  The finding *refines* CMOC 13 by specifying the *nuances* of this abandonment. It's not just a general feeling of being forgotten; it includes feeling *unimportant*, like their condition isn't a priority, and a sense of *stasis* ("nothing's ever going to get done"). This suggests the follow-ups need to do more than just exist; they need to actively counteract these specific feelings. It implies that the *content* and *quality* of the interactions are crucial. They need to reassure patients that they *are* remembered that their condition *is* being considered, and that their care *is* progressing, even if slowly. It's not just about *contact*, but *meaningful and supportive* contact.  Therefore, this finding strongly supports CMOC 13 by demonstrating the negative psychological impact of long wait times. It also *refines* the theory by highlighting the specific feelings of being forgotten, unimportant, and stuck, emphasizing the need for *meaningful and supportive* contact that goes beyond simply checking in. The refined theory might suggest that regular contact (e.g., telephone calls, video messages, personalized letters) from healthcare professionals should not only mitigate feelings of abandonment but also actively address the psychological impact of waiting, reassuring patients that they are remembered, valued, and that their care remains a priority.  **CMOC 3**  p.142 *“Our study also highlighted a gap in engaging a patient– centred approach with patients reporting not being asked specifically about their goals. Ascertaining what is meaningful to patients in the preoperative, perioperative and postoperative periods may be challenging but is fundamental to executing patient-centred care in practice as engaging patients in their care has been associated with improved clinical outcomes and care experience”*  This finding *strongly supports* and *refines* CMOC 3. The theory argues that patient choice in goal setting (C) triggers agency, empowerment, and motivation (M), leading to increased engagement (O). The finding about patients reporting not being asked specifically about their goals directly points to a *gap* in implementing this principle of patient-centred care. It underscores the importance of actively soliciting patient input and understanding their individual goals.  The finding *refines* CMOC 3 in several ways:   - **It highlights the challenge of *eliciting* patient goals:** The phrase "ascertaining what is meaningful to patients...may be challenging" acknowledges that it's not always easy to understand what truly motivates each individual. It suggests that healthcare professionals need specific strategies and training to effectively elicit patient goals. - **It broadens the scope of "goals" beyond just "exercise":** The finding refers to goals in the "preoperative, perioperative and postoperative periods," suggesting that patient goals can encompass a wider range of concerns than just the exercise component of prehabilitation. It implies that patients may have goals related to pain management, emotional well-being, functional recovery, or other aspects of their surgical journey. - **It connects patient-centred care to improved outcomes:** The finding explicitly links engaging patients in their care to "improved clinical outcomes and care experience." This reinforces the importance of patient choice and agency, not just for motivation, but also for tangible improvements in health and well-being.   Therefore, this finding strongly supports CMOC 3 by highlighting the importance of actively engaging patients in goal setting. It also *refines* the theory by acknowledging the challenges of eliciting patient goals, broadening the scope of "goals" beyond exercise, and explicitly linking patient-centred care to improved outcomes. The refined theory might suggest that prehabilitation programs should incorporate structured processes for eliciting patient goals across the surgical journey, recognizing that these goals may extend beyond exercise and that actively engaging patients in this process can lead to better outcomes and experiences.  **Current CMOC 3:**  When patients are given the choice to decide their own goals for the exercise component of a prehabilitation program (C), it can trigger feelings of agency, empowerment, and motivation (M). This sense of ownership can make the programme more meaningful and increase engagement, as patients are actively involved in shaping their prehabilitation programme (O).  **Refined CMOC 3:**  When patients are *actively engaged in identifying and defining their own personally meaningful goals* for their prehabilitation program, *using structured and supportive processes facilitated by healthcare professionals* (C), it can trigger feelings of agency, empowerment, and motivation (M). This sense of ownership, *combined with a clear understanding of how these goals relate to improved prehabilitation outcomes*, can make the program more meaningful and increase engagement, as patients are actively involved in shaping their prehabilitation program (O).  **Key Changes and Rationale:**   - **"*actively engaged in identifying and defining their own personally meaningful goals*..."**: This emphasizes the *active* role patients should play in defining their goals. It also highlights the importance of the goals being *personally meaningful* to the individual. - **"*using structured and supportive processes facilitated by healthcare professionals*..."**: This highlights the need for *structured processes* to elicit patient goals and the importance of *healthcare professionals facilitating* this process. It acknowledges that patients may need guidance and support in identifying and articulating their goals. - **"*combined with a clear understanding of how these goals relate to improved prehabilitation outcomes*..."**: This adds the crucial element of *understanding the connection* between the chosen goals and the desired *prehabilitation* outcomes. Patients are more likely to be motivated if they understand how their goals will contribute to their prehabilitation progress.   The refined CMOC 3 emphasizes the importance of *actively engaging* patients in defining *personally meaningful goals* for their *prehabilitation program*. It highlights the need for *structured processes* and *professional facilitation* to support this goal-setting process. It also underscores the importance of connecting these goals to *improved prehabilitation outcomes* to enhance motivation and engagement. |
| --- |
